# Synthesis and Characterization of Novel Polythiophenes Containing Pyrene Chromophores: Thermal, Optical and Electrochemical Properties

**DOI:** 10.3390/molecules21020172

**Published:** 2016-01-30

**Authors:** Bianca X. Valderrama-García, Efraín Rodríguez-Alba, Eric G. Morales-Espinoza, Kathleen Moineau Chane-Ching, Ernesto Rivera

**Affiliations:** 1 Instituto de Investigaciones en Materiales, Universidad Nacional Autónoma de México, Circuito Exterior Ciudad Universitaria, Mexico City 04510, Mexico; biancaxvg@msn.com (B.X.V.-G.); efrainhelio@gmail.com (E.R.-A.); erichsm536@yahoo.com.mx (E.G.M.-E.); 2 Centre National de la Recherche Scientifique (CNRS), Laboratoire de Chimie de Coordination (LCC), 205, Route de Narbonne, Toulouse F-31077, France; 3 Institut National Polytechnique, Université de Toulouse, Université Paul Sabatier, Toulouse F-31077, France

**Keywords:** polythiophene, pyrene, electrosynthesis, absorption, fluorescence

## Abstract

A novel series of pyrene containing thiophene monomers **TPM1**–**5** were synthesized and fully characterized by FTIR, MS, ^1^H- and ^13^C-NMR spectroscopy; their thermal properties were determined by TGA and DSC. These monomers were chemically polymerized using FeCl_3_ as oxidizing agent to give the corresponding oligomers **TPO1**–**5**) and they were electrochemically polymerized to obtain the corresponding polymer films deposited onto ITO. All oligomers exhibited good thermal stability, with T_10_ values between 255 and 299 °C, and T_g_ values varying from 36 to 39 °C. The monomers showed an absorption band at 345 nm due to the S_0_ → S_2_ transition of the pyrene group, whereas the fluorescence spectra showed a broad emission band arising from the “monomer” emission at 375–420 nm. The obtained polymers exhibited two absorption bands at 244 and 354 nm, due to the polythiophene and the pyrene moieties, respectively. The fluorescence spectra of polymers showed a broad “monomer” emission at 380–420 nm followed by an intense excimer emission band at 570 nm, due to the presence of intramolecular pyrene-pyrene interactions in these compounds.

## 1. Introduction

In recent years, the synthesis and characterization of polymers containing heteroaromatic rings have been widely studied because of their potential in advanced optoelectronic applications [1,2,3,4,5]. Polythiophene (PT) has been considered one of the most promising π-conjugated polymers due to its high stability, ease of structural modification and controllable optical and electrochemical properties. At the beginning the applications of non-substituted polythiophene were very limited because of its insolubility in many organic solvents, due to its extended π-conjugated structure. Furthermore, alkyl chains have been incorporated into the thiophene units in order to obtain functional monomers able to yield soluble polymers. The poly(3-alkylthiophene)s resulted to be highly processable conducting polymers, whose solubility allowed their full characterization by spectroscopic methods [6]. The stability of poly(3-alkylthiophene)s in the doped state can be performed by introducing alkoxy chains. The incorporation of alkoxy groups into polythiophenes increases significantly their conductivity without decreasing their solubility in many organic solvents [7,8].

Most polythiophenes exhibit interesting optical properties such as thermochromism [9,10,11], ionochromism [10,11,12], photochromism [13], piezochromism [14] and biochromism [15]. Colour changes are mainly due to transitions from planar to twisted conformation of the polymer backbone and *vice versa*, which modifies the conjugation length, thereby causing a shift of the absorption bands in their UV-vis spectra [16]. In polymers containing alkoxy groups at 3-position of the thiophene rings, the lone pairs of the oxygen atom enter into conjugation with the polythiophene backbone, inducing a planar conformation, electronic mobility and a higher conductivity [17].

The chromic properties of substituted polythiophenes make them excellent prospects for light emitting diodes [18], gas sensors [19,20,21], biomedical applications [22], metal ions sensors [23,24], biosensors [25,26,27,28], and other related applications [29,30,31].

On the other hand, pyrene is a very useful fluorescent probe which has been widely employed for polymer labeling because it easily forms excimers. Moreover, pyrene exhibits a longer singlet lifetime than other chromophores, which favors the formation of excimers. The main photophysical properties of pyrene excimers have been discussed in detail by Winnik [32]. An excimer emission band appears if a pyrene molecule in the excited state associates with another in the ground state, after which a photon is delocalized over the conjugate to show a net change in the fluorescence spectrum profile. The resulting optical and photophysical properties provide us useful information about the molecular geometry, internal stacking of the pyrene groups and polymer interactions at long distance.

In our research group, we have synthesized and characterized a wide variety of π-conjugated polymers and oligomers containing pyrene units in their structure. We studied the effect of the internal stacking of the pyrene groups on the optical and photophysical properties [33,34,35,36,37]. Very recently, we carried out the incorporation of pyrene moieties into polythiophenes in order to obtain donor-acceptor systems for optical applications [29,30,31,38,39]. These polymers can be synthesized either by chemical or electrochemical methods. In fact, the electropolymerization offers many advantages over other synthetic approaches, such as the absence of catalyst, a direct deposition of a doped polymer film onto the electrode surface, *in situ* characterization and easy control of the thickness of the deposited film [14].

**Figure 1:**
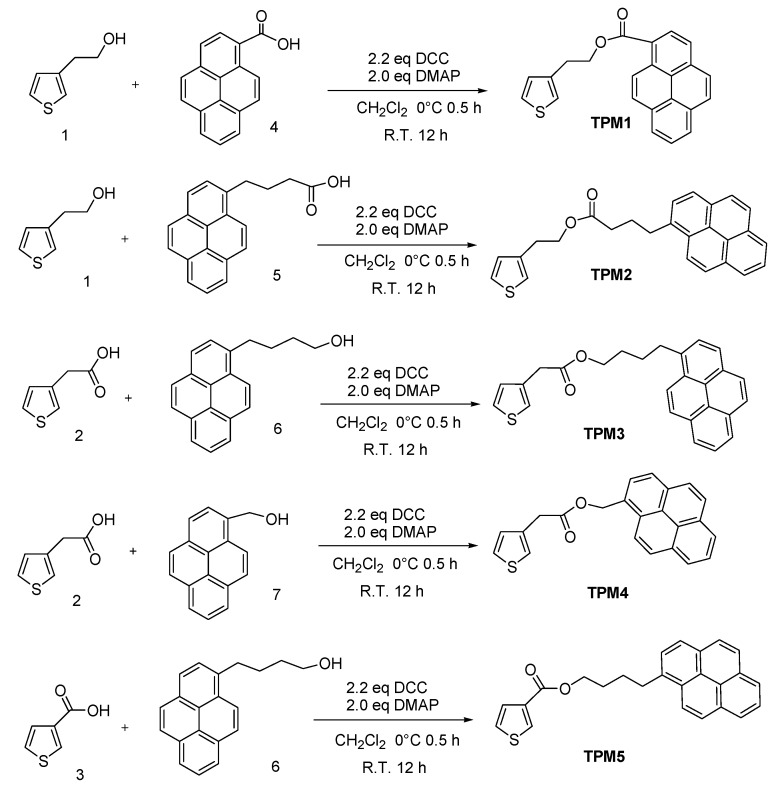
Synthesis of thiophene monomers containing pyrene groups.

**Figure 2:**
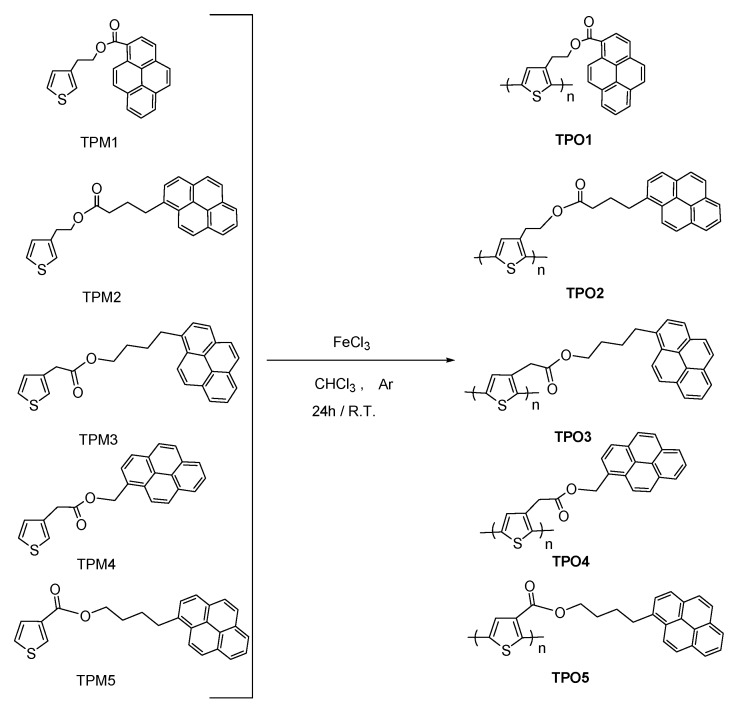
Synthesis of the obtained oligothiophenes, where the n values for **TPO1**–**TPO5** are 6, 8, 6, 5, and 8 respectively.

**Figure 3:**
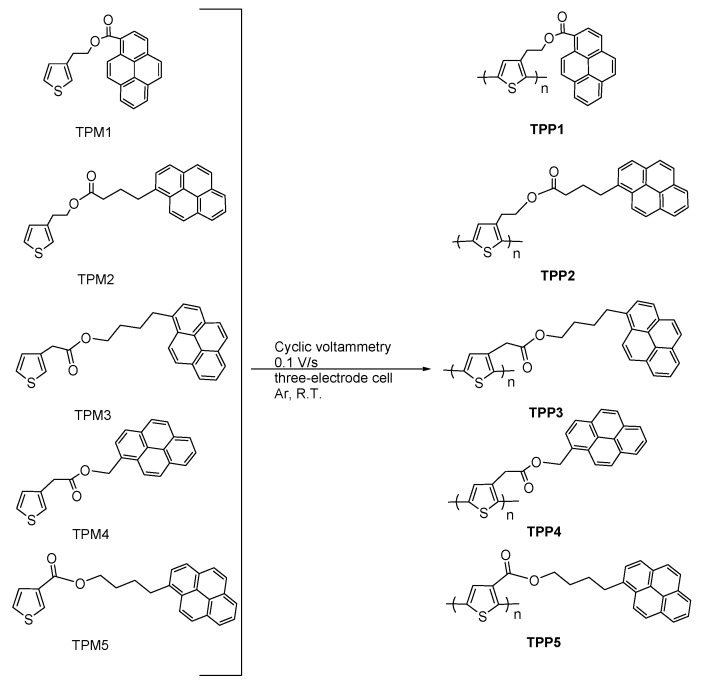
Electrochemical synthesis of polythiophenes containing pyrene units.

In this work, we report the synthesis and characterization of a novel series of thiophene monomers **TPM1**–**5** containing pyrene units and alkyl spacers linked by an ester group (Figure 1), which were chemically polymerized to give the corresponding oligothiophenes (Figure 2) and electrochemically polymerized to form polythiophenes (Figure 3). Monomers were fully characterized by FTIR, MS-CI, ^1^H- and ^13^C-NMR spectroscopy. In **TPM1** the carbonyl group is directly attached to the pyrene unit, in **TPM5** to the thiophene ring and in the other monomers (**TPM2**, **TPM3** and **TPM4**) the ester function is located in the middle of the flexible spacer, in order to study the effect of the position of the carbonyl group on the optical and electronic properties. The thermal properties were determined by TGA and DSC, and the optical properties were studied by UV-Vis and fluorescence spectroscopy. Regarding the oligomers, these compounds were characterized by MALDI-TOF, and their thermal properties were analyzed by TGA and DSC. Unfortunately, it was not possible to characterize them by NMR in solution because they resulted to be insoluble in many organic solvents, which can be due to the presence of the pyrene units that undergo the formation of aggregates. A similar behavior has been observed in other polymers with high pyrene content [38,40]. Concerning the electrochemically obtained polymers, they were studied by cyclic voltammetry in order to determine their oxidation and polymerization potentials. The optical properties of the obtained polythiophenes were studied by absorption and fluorescence spectroscopies in solid state. Due to their chromic properties, pyrene’s ability to form excimers and oxygen sensitivity, these oligomers and polymers can be used as sensors or as luminescent materials in OLEDs [41,42,43].

## 2. Results and Discussion

### 2.1. Spectroscopical Characterization

**Figure 4:**
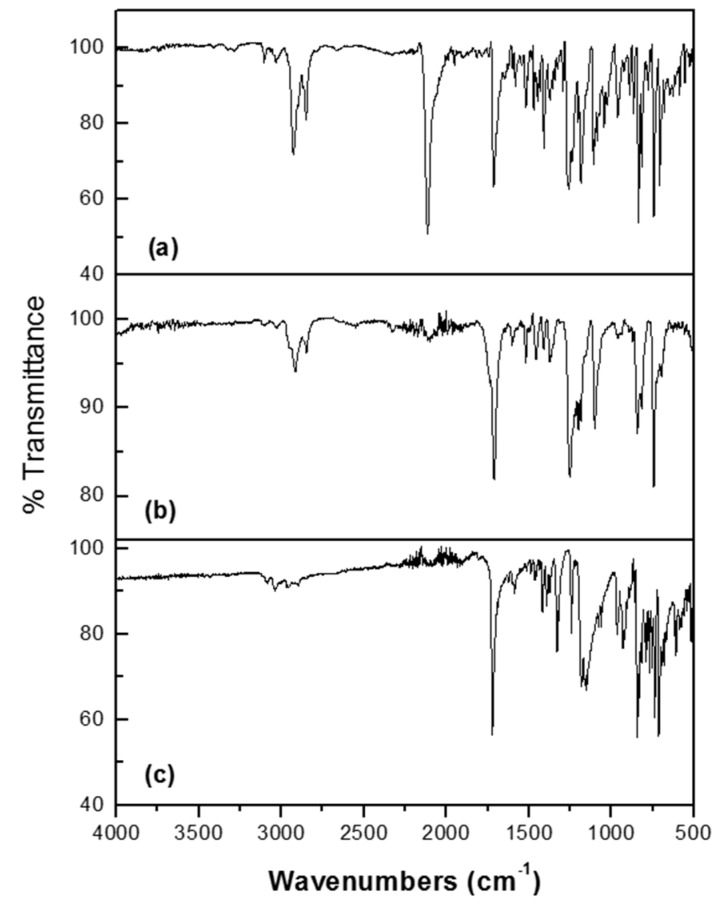
FTIR spectra (**a**) monomer **TPM5**; (**b**) oligomer **TPO5** and (**c**) polymer **TPP5**.

The obtained monomers were characterized by FTIR and NMR spectroscopies. In the FTIR spectra of these compounds (Figure 4), we can observe two bands at 3106 and 3026 cm^−1^ due to the C-H bonds of the pyrene unit and the thiophene ring, respectively. Moreover, we can perceive two bands at 2930 and 2850 cm^−1^ due to asymmetric vibration of CH_2_ groups, as well as another band at 1723 cm^−1^ attributed to the C=O bond (stretching) of the ester, followed by a band at 1643 cm^−1^ corresponding to C=C bonds of the aromatic rings. In addition, we can see two bands at 1255 and 1197 due to C-O bond of the ester. Finally, two prominent bands at 838 and 742 cm^−1^ arising from C-H (out of plane) were also observed.

The ^1^H-NMR spectra of monomers in CDCl_3_ solution are shown in the Supplementary Materials (Schemes S1–S5 in Supplementary Materials). As we can see, all spectra show a multiplet at *ca.* 7.85–9.20 ppm, due to the protons of the pyrene unit, followed by a double doublet at 7.51 ppm with *J* = 4.6 Hz assigned to proton H^5^ of the thiophene ring. A doublet at 7.21 ppm, attributed to proton H^2^ with *J* = 4.7 Hz and a double doublet at 7.04 with *J* = 4.9 Hz corresponding to proton H^4^ of thiophene were also observed. In the aliphatic region, we can perceive the signals due to protons of the CH_2_ groups of the aliphatic segment and those arising from the protons of OCH_2_ groups, which appear between 3–4 ppm.

In the ^13^C-NMR spectra of monomers, we can notice four signals at 155, 138, 118 and 96 ppm, due to carbons of the thiophene ring C^2^, C^3^, C^4^, and C^1^, respectively. In the aromatic region, we observed the signals corresponding to carbons of pyrene unit (16 carbons), which appeared between 134–120 ppm. The signal of OCH_2_ protons can be seen at 65 ppm, as well as a series of peaks between 28–36 ppm attributed to the methylene groups CH_2_-CH_2_ of the aliphatic chain.

All monomers were chemically polymerized using FeCl_3_ as oxidizing agent to give the corresponding oligomers and electrochemically polymerized to give a series of polymers, which were characterized by FTIR. The polymerization reaction conditions are described in the experimental section. The FTIR spectra of **TPM5**, **TPO5**, and **TPP5** are illustrated in Figure 4. The polymerization was confirmed by FTIR spectroscopy; the obtained oligomers and polymers exhibited the same bands as their precursor monomers. However, we can observe a decrease in intensity of the bands at 3106 and 3026 cm^−1^ due to the disappearance of the C-H bonds present in the thiophene ring of the monomer to form the new C-C bonds of the polymer, which confirmed that the polymerization reaction took place. Moreover, the bands at 838 and 742 cm^−1^ (C-H, out of plane) diminished in intensity.

### 2.2. Thermal Properties

The thermal stability of the compounds was determined by thermogravimetric analysis (TGA). The melting point as well as the glass transition temperature (T_g_) were measured with a Differential Scanning Calorimeter (DSC). The thermal properties of monomers and oligomers are summarized in Table 1.

**Table 1:** Thermal properties of monomers and oligomers.

Compound	m.p. (°C)	Compound	m.p. (°C)/T_g_ (°C)	T_10_ (°C)	Degradation (°C)	% W_t_ Remaining
**TPM1**	117.6	**TPO1**	>250/38.0	292.6	310–348	3.33
**TPM2**	66	**TPO2**	>250/36.0	299.3	310–330	12.16
**TPM3**	83.3	**TPO3**	>250/39.1	272.7	277–347	12.14
**TPM4**	67.7	**TPO4**	>250/36.1	255.9	260–315	38.71
**TPM5**	96	**TPO5**	>250/37.9	273.2	313–374	4.659

Concerning the melting points of the monomers, we can notice that the melting point of **TPM1** is higher than that of the others compounds, this might be due to a structural arrangement or stacking of the pyrene units, which provides higher crystallinity to the molecule. In the case of the oligomers, we can observe that their T_g_ values are very similar (between 36 and 39 °C), and they are thermally stable above 250 °C.

**Figure 5:**
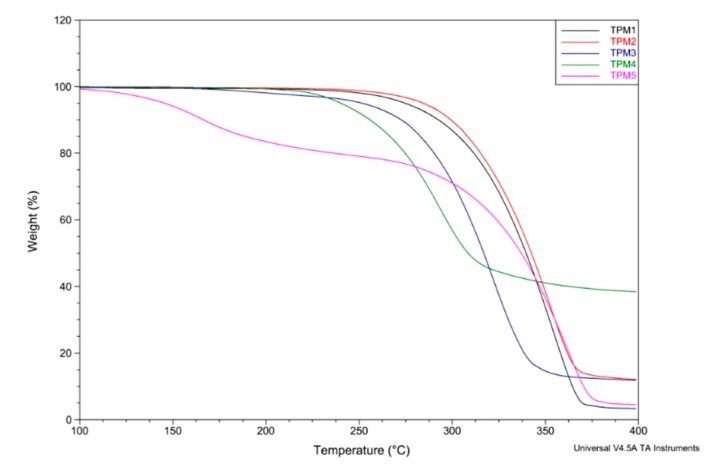
TGA curves of the oligomers **TPO1**–**5**.

TGA thermograms of the obtained oligomers are shown in Figure 5. The decomposition profile of these compounds are very similar to that reported for polythiophene itself [44], leaving between 3.3–38.7 wt % of remains after having heated up to 400 °C. This can be due to the rigidity of the **TPO1**–**5** backbone. The thermograms exhibited drastic degradation above 260 °C with T_10_ values between 256–299 °C. Similarly, **TPO1** and **TPO2** resulted to be thermally more stable with T_10_ values between 293–299 °C, showing degradation beyond 300 °C.

### 2.3. Optical Properties of the Monomers

**Figure 6:**
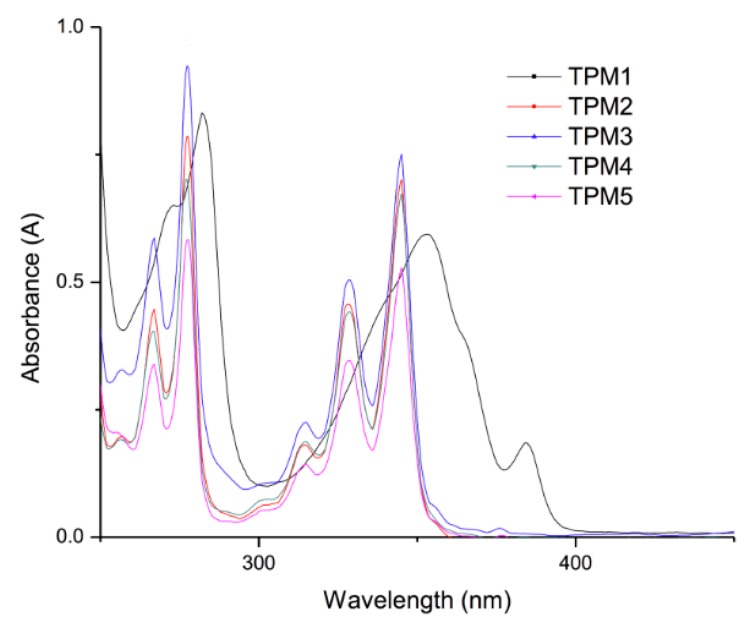
Absorption spectra of the monomers in CH_2_Cl_2_ solution (conc. 1.0 × 10^−5^ M).

**Figure 7:**
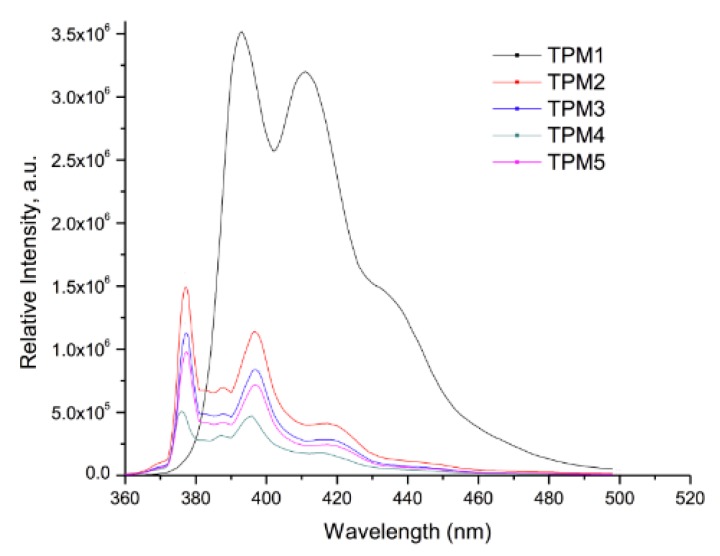
Emission spectra of the monomers in CH_2_Cl_2_ solution (conc. 5 × 10^−7^ M).

The optical properties of the monomers were studied in fresh CH_2_Cl_2_ solution by absorption and fluorescence spectroscopy; the results are summarized in Table 2. The UV-vis and fluorescence spectra of the monomers are shown in Figure 6 and Figure 7, respectively. It has been verified that the Beer-Lambert law applies for the used concentrations. For all compounds, a pyrene concentration of 5.0 × 10^−7^ M was used, the slit widths of the excitation and emission monochromators were equaled to 2 and 1 nm, respectively.

**Table 2:** Optical properties of the monomers.

Compound	Absorption (λ nm)	Cut off (nm)	Emission (nm)	Cut off (nm)
**TPM1**	353	450	391–440 ^a^	500
**TPM2**	345	450	377–416 ^a^	500
**TPM3**	345	450	377–416 ^a^	500
**TPM4**	345	450	377–416 ^a^	500
**TPM5**	345	450	377–416 ^a^	500

^a^ “Monomer” emission.

The absorption spectra of the monomers in CH_2_Cl_2_ solution (Figure 6) exhibited a well-structured absorption band at λ = 345 nm, due to the S_0_ → S_2_ transition of the pyrene units. Since the S_0_ → S_1_ transition band of this chromophore has a very low intensity and appears at 375 nm, for practical purposes all monomers were excited at λ = 345 nm in order to get good quality emission spectra. As we can observe, monomer **TPM1** exhibited a red shifted S_0_ → S_2_ absorption band (λ = 353 nm), due to the electron-withdrawing effect of the carbonyl group directly linked to the pyrene unit. In addition, this band shows a significant broadening, which reveals the presence of intramolecular pyrene-thiophene interactions in the ground state. This can be attributed to the short alkyl spacer and the presence of the carbonyl group linked to pyrene. All these monomers exhibited molar extinction coefficient values of ε = 39,000 M^−1^·cm^−1^ at λ = 345 nm, very similar to that reported for pyrene itself in THF solution (ε = 45,000 ± 10,000 M^−1^·cm^−1^).

The fluorescence spectra of the monomers are shown in Figure 7. As we can see, all compounds exhibited a broad “monomer” emission band at 377–440 nm, which arises from the S_1_ → S_0_ transition of pyrene in the non-associated state [32]. It is worth to point out that monomer **TPM1** shows a higher intensity emission band compared to those of the other monomers. Besides, this monomer exhibits emission beyond 450 nm, a wavelength where pyrene does not emit (discrete excimer emission). Taking into account these results jointly with those obtained by absorption spectroscopy, we can affirm that we have the presence of intramolecular pyrene-thiophene interactions in this monomer. Except for **TPM1**, no excimer emission was observed for the other monomers. It is evident that the optical properties of these monomers are mainly dominated by the pyrene unit.

### 2.4. Optical Properties of the Polymers

**Figure 8:**
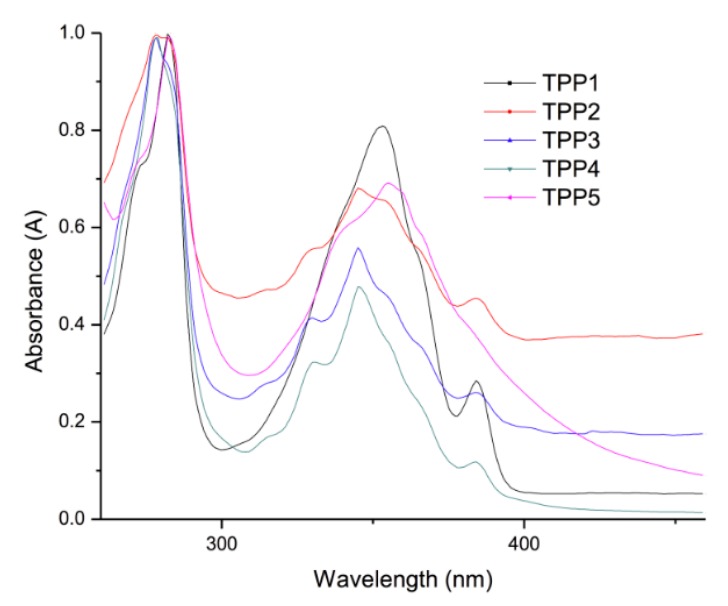
Normalized absorption spectra of the polymers in thin film deposited on ITO/glass.

**Figure 9:**
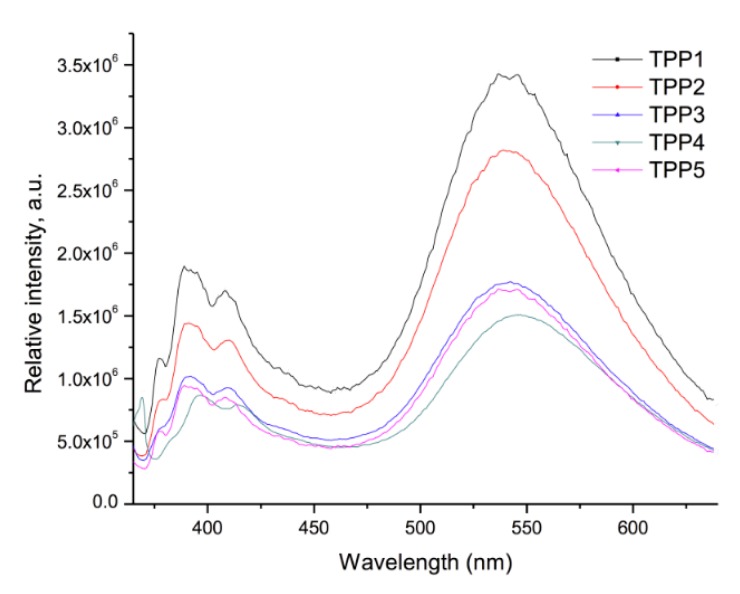
Emission spectra of the polymer films deposited on ITO/glass.

The optical properties of the oligomers were also studied by absorption and fluorescence spectroscopy in solid state. For that purpose, thin films were prepared with the oligomers by deposition of CH_2_Cl_2_ solutions on ITO/glass plates and the samples were excited at λ_exc_ = 360 nm. The results are summarized in Table 3. The UV-vis and emission spectra of the polymers are shown in Figure 8 and Figure 9, respectively.

**Table 3:** Optical properties of the polymers deposited on ITO/glass.

Compound	Absorption (λ nm)	Cut off (nm)	Emission (λ nm) λ_exc_ = 360 nm	Cut off (nm)
**TPP1**	275 ^b^–356 ^a^	450	388 ^c^, 409 ^c^, 545 ^d^	640
**TPP2**	273 ^b^–345 ^a^	450	391 ^c^, 410 ^c^, 539 ^d^	640
**TPP3**	273 ^b^–345 ^a^	450	389 ^c^, 409 ^c^, 543 ^d^	640
**TPP4**	274 ^b^–345 ^a^	450	397 ^c^, 413 ^c^, 543 ^d^	640
**TPP5**	275 ^b^–357 ^a^	450	390 ^c^, 409 ^c^, 544 ^d^	640

^a^: absorption band of the pyrene; ^b^: absorption band of the polythiophene backbone; ^c^: monomer emission; ^d^: excimer emission.

The absorption spectra of polymers **TPP1**–**5** are illustrated in Figure 8. As we can see, all polymers exhibited an absorption band at *ca.* λ = 245 nm corresponding to S_0_ → S_1_ (n-π) transition of the polythiophene backbone, which adopts a semi-twisted conformation, followed by a broad band at λ = 350 nm due to the S_0_ → S_2_ transition of the pyrene groups. This broadening is a clear indication of the existence of intramolecular pyrene-pyrene interactions in the polymers, which was further confirmed by fluorescence spectroscopy. On the other hand, the emission spectra of the polymers (Figure 9) showed a well structured “monomer” emission band at *ca.* λ = 390–410 nm followed by a very intense broad excimer band at λ = 545 nm, which reveals the presence of intramolecular pyrene-pyrene interactions. It is evident that the alkyl spacers jointly with the coiling of the polymer backbone allow pyrene units to encounter even at long distance.

### 2.5. Electrochemical Properties of Monomers and Polymers

**Figure 10:**
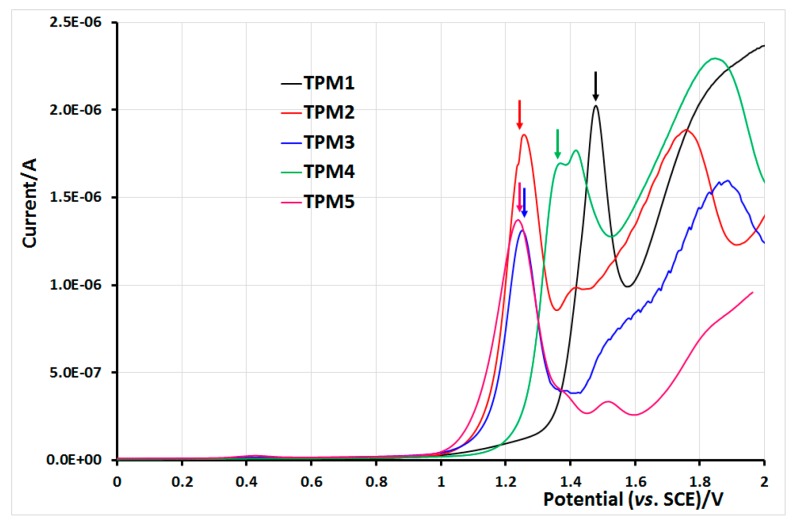
Square wave voltammograms of the monomers in 0.1 M TBA·PF6, CH_2_Cl_2_, Pt electrode, *vs.* SCE, scan speed 100 mV/s. Peaks relative to pyrene oxidation are indicated by thin arrows.

During oxidation of the monomers, firstly we can observe the reversible oxidation peak of pyrene as reported in the literature [45]. At higher potential, the thiophene moiety was irreversibly oxidized leading to a classical electropolymerization due to a coupling process of the heterocycles (Figure 10). This was evidenced by square wave voltammetry (SWV), whose main advantage is an excellent sensitivity and the rejection of background currents. For each monomer, we can observe a narrow SWV signal of the reversible oxidation of the pyrene unit, followed by a larger signal due to irreversible oxidation of thiophene. The influence of the molecular structure on the potential oxidation value is illustrated in Figure 10. In monomers **TPM2**, **TPM3** and **TPM5**, the pyrene moiety is linked to the ester function via an alkyl chain bearing from 3 to 4 carbon atoms. In that case, the pyrene oxidation was recorded at E1/2 = 1.25 V/SCE and the thiophene oxidation was measurable from 1.4 V/SCE. In monomer **TPM1**, the pyrene moiety was in direct conjugation with the ester function, which resulted in a shift of the oxidation potential value of pyrene to a higher potential about E1/2 = 1.48 V/SCE. At this potential, the oxidation of thiophene was initiated and developed more largely above 1.6 V. Monomer **TPM4** showed an intermediate situation with only one carbon atom between the pyrene ring and the ester function. Here, the pyrene unit was oxidized at E_1/2_ = 1.35 V/SCE; immediately above this potential, the oxidation of thiophene can be seen.

**Figure 11:**
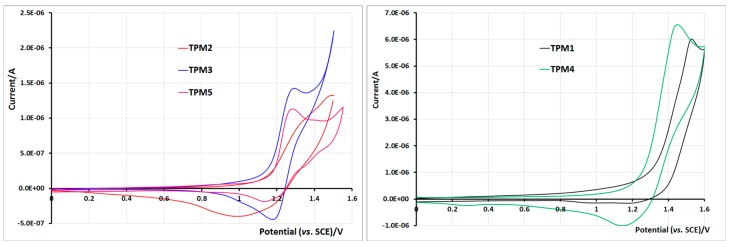
Cyclic voltammetry of monomers **TPM2**, **TPM3** and **TPM5** (**left**) and monomers **TPM1** and **TPM4** (**right**) in 0.1 M TBA·PF_6_, CH_2_Cl_2_, Pt electrode, *vs.* SCE, scan speed 200 mV/s.

The cyclic voltammogram (CV) under standard conditions (0.1 M TBA·PF_6_, CH_2_Cl_2_, Pt, SCE, scan speed 200 mV·s^−1^) exhibited several oxidation features. For **TPM2**, **TPM3** and **TPM5**, on the first scan, we could distinguish the oxidation signal of pyrene followed by that of thiophene. As in the previous case, the oxidation of pyrene occurred at the same potential of 1.25 V/SCE (Figure 11 left). When the sweep was pursued until 1.5–1.55 V/SCE, we could see the oxidation signal of thiophene. For **TPM2**, where the thiophene ring being attached to the ester function through a 2-carbon linker, the ester function had a weak influence on the oxidation potential of the heterocycle ring, which was oxidized just after the pyrene moiety, so that both oxidation signals are fused (see the red curve in Figure 11 left). On the contrary, in monomer **TPM5**, the thiophene ring was in direct conjugation with the ester function. The withdrawing effect of the ester resulted in a higher oxidation potential of the thiophene ring as demonstrated by the corresponding voltammogram (pink) (Figure 11, left).

The situation of monomer **TPM3** is intermediate, with only one carbon atom between the thiophene and the ester function. Its voltammogram exhibited an in-between behavior in comparison to the two preceding cases: the reversible oxidation of pyrene was easily observed and immediately followed by the oxidation of the thiophene ring. For the monomers **TPM1** and **TPM4**, the two oxidation waves of pyrene and thiophene, respectively, were more difficult to distinguish. Since the oxidation potential of pyrene was close to that of thiophene, the corresponding waves overlapped (Figure 11 right).

**Figure 12:**
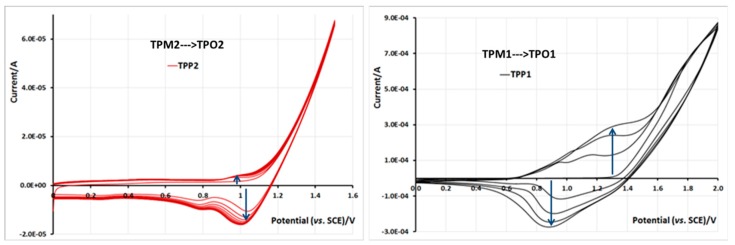
Influence of the potential range on the thickness and electroactivity of the formed film in 0.1 M TBA·PF_6_ and 5 × 10^−3^ M of **TPM2** (**left**) and **TPM1** (**right**) in CH_2_Cl_2_, ITO electrode, *vs.* SCE, scan speed 100 mV/s, 6 and 4 first cycles, respectively.

Homogeneous films of the different polymers were easily synthesized on ITO/glass electrodes in the potentiodynamic mode with 0.1 M TBA·PF_6_ dichloromethane solutions of monomers (5 × 10^−3^ M). In each case, the film generated at 100 mV/s exhibited good electroactivity. To obtain good quality films, the potential range is of significant importance: for example, monomers **TPM1** and **TPM2** showed similar behaviors during the electropolymerization since in both cases the thiophene ring is linked to the ester function by a 2-carbon chain. While the potential sweep was set between 0 and 1.5 V/SCE we could perceive a weak redox signal due to the deposition of a very thin film of low electroactivity (see Figure 12 left). When the upper limit was fixed at 2 V/SCE, we observed the development of a perfectly electroactive film in the 0.4–1.5 V/SCE range (Figure 12 right). The growing value of current from cycle to cycle is related to the progressive growing of the electroactive film thickness.

**Figure 13:**
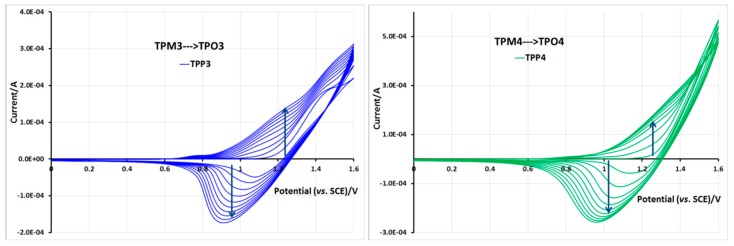
Film formation during cycling in 0.1 M TBA·PF6 and 5 × 10^−3^ M of TPM3 (**left**) and TPM4 (**right**) in CH_2_Cl_2_, and electroactivity of the corresponding polymer films. ITO electrode, potential *vs.* SCE, scan speed 100 mV/s. 10 first cycles.

Monomers **TPM3** and **TPM4** behaved similarly since their thiophene units are separated from the ester function by only one carbon atom (Figure 13). In both cases, 1.6 V/SCE as the upper potential limit is a suitable value to obtain satisfactory growing films. The current values increased regularly from one cycle to another and the films exhibited electroactivity between 0.8 and 1.2 V/SCE that means, in a narrower range than for the two preceding polymers.

**Figure 14:**
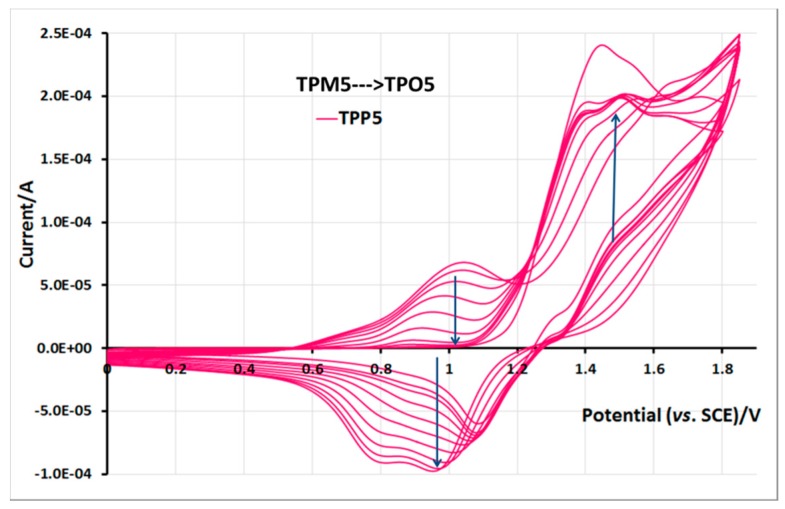
Film formation during cycling in 0.1 M TBA·PF_6_ and 5 × 10^−3^ M of TPM5 in CH_2_Cl_2_, and electroactivity of the corresponding polymer film. ITO electrode, potential *vs.* SCE, scan speed 100 mV/s. 10 first cycles.

For monomer **TPM5**, the potential sweep must be pursued up to 1.8 V/SCE to get good quality films; this high potential is necessary because of the electro-withdrawing effect of the ester function directly linked to the thiophene ring. In the voltammogram (Figure 14) recorded during the electropolymerization, we can clearly see the electroactivity of the formed polythiophene in the range of 0.6–1.2 V/SCE, then we can distinguish the redox signal of the pyrene group at *ca.* 1.0–1.5 V/SCE, and at higher potential the oxidation signal of the thiophene ring. It should be noted that, despite the high oxidation potential value of thiophene, the formed film exhibits a very good electroactivity, thereby showing a good stability towards redox phenomena. These changes can be macroscopically seen by the color change of the polymer films in neutral state, which have a color between transparent and light yellow, whereas the oxidized polymer films adopt a color between yellow and orange.

The most important point here is that, for all monomers, even if pyrene was submitted to redox process during the formation of the film, this chromophore did not interfere in the electropolymerization process. This feature allowed us to synthesize polythiophene films bearing pyrene units on surface by electrochemical means. Since the electroactivity potential range of the polythiophene ring is very different of that of pyrene, the polymers can be held in their oxidized or neutral state without affecting the redox state of pyrene.

To clarify this point we made a comparison between the voltammograms of the TPM polymers and non-substituted pyrene [46]. This chromophore shows two oxidation anodic peaks at 1.338 (middle intensity) and 1.572 V (intense) with higher potential values than those found for our synthesized monomers **TPM2**–**4** (1.25 V), where the pyrene unit is not directly linked to the ester. By contrast, in **TPM1** the pyrene oxidation potential was shifted to 1.48 because it is directly attached to the ester. The cyclic voltammetry of non-substituted pyrene shows also two discrete anodic reduction peaks at 0.616 and 0.790 V, whereas TPM monomers exhibit more intense catodic reduction peaks between 0.8 and 1.2 V. By comparing the cyclic voltammograms of the TPM monomers with that of pyrene itself, we confirm that the thiophene moiety exhibits higher electroactivity towards polymerization.

## 3. Experimental Section

### 3.1. Apparatus

FTIR spectra of the monomers and polymers were recorded on a 510 P spectrometer (Nicolet, Madison, WI., USA) in solid state. ^1^H- and ^13^C-NMR spectra of all compounds were carried out in CDCl_3_ solution, in a Bruker Avance 300 spectrometer (Bruker, Coventry, UK), operating at 300 MHz and 75 MHz for ^1^H and ^13^C, respectively. Molecular weights of the monomers were determined by mass spectrometry using a chemical ionization technique in a Bruker-Microflex spectrometer (Bruker, Coventry, UK).

For the oligomers, the molecular weights were determined by MALDI-TOF^+^ in positive mode, using trans-2-[3-(4-t-butyl-phenyl)-2-methyl-1,2-propenylidene]malonitrile (DCTB) as matrix in a Bruker-Microflex mass spectrometer. The mass spectrometry analysis showed the molecular ion peaks [M^+^] with the corresponding *m*/*z* values for each molecule.

The thermal stability of the compounds was determined by thermogravimetric analysis (TGA) and conducted on a Hi-Res TGA 2950 Instrument (TA Instruments, New Castle, DE, USA). The melting point of monomers and oligomers as well as the glass transition temperature (Tg) were measured with a Differential Scanning Calorimeter (MDSC) TA Instruments 2920 (TA Instruments).

Absorption spectra of the monomers in CH_2_Cl_2_ solution (spectrometric grade, concentration 1 × 10^−5^ M) were recorded on a Varian Cary 1 Bio UV-vis spectrophotometer (Agilent Technologies, Les Ulis, France) model 8452A. Fluorescence spectra corrected for emission detection were recorded on a LS-100 steady-state instrument (Photon Technology International, London, ON, Canada) equipped with a continuous UXL-75Xe Xenon arc lamp (Ushio, Cypress, CA, USA) and a PTI 814 photomultiplier detection system. The slit widths on the excitation and emission monochromators were equaled to 2 and 1 nm, respectively, for the monomers, and 4 and 9 for the polymers. Each solution was excited at λ = 345 nm using a 1 cm quartz cell. For all monomers, a concentration of 5 × 10^−7^ M was employed in order to obtain an absorbance value of 0.05 at λ = 345 nm, to avoid any inner filter effect [47].

Cyclic voltammetry measurements were performed with an Autolab PGSTAT100 potentiostat controlled by GPES 4.09 software (HORIBA Jobin Yvon SAS, Longjumeau, France). All experiments were carried out at room temperature (298 ± 2 K), in a homemade airtight three-electrode cell connected to a vacuum/argon line. Cyclic voltammograms were recorded at a scan rate of 100 mV/s on Pt and the obtained polymers were deposited on ITO on glass plates (0.8 cm × 5.0 cm) at 200 mV/s. ITO/glass plates were purchased from SOLEMS and consisted of 100 nm thickness ITO (25–35 ohms) deposited on glass (1.1 mm thick).

### 3.2. Chemicals

*N*,*N*′-Dicyclohexylcarbodiimide (DCC, 99%) and 4-dimethylaminopyidine (DMAP) (99%), 1-pyrenemethanol, 3-thiopheneacetic acid 98%, 3-thiopheneethanol, 1-pyrenebutyric acid, 1-pyrenecarboxylic acid 98%, thiophene carboxylic acid, and FeCl_3_ were purchased from Sigma Aldrich (Mexico City, Mexico) and used as received. Dichloromethane (CH_2_Cl_2_), n-hexane and chloroform (CHCl_3_) were purchased from Tecsiquim (Mexico City, Mexico). Chloroform, used in the polymerizations was distilled over calcium hydride (CaH_2_) in order to remove traces of water.

### 3.3. Electrochemical Polymerization

A saturated calomel electrode (SCE) separated from the solution by a bridge compartment was used as the reference electrode. The counter electrode was a platinum wire of *ca.* 1 cm^2^ apparent surface. For electrochemical examination of the monomers, the working electrode was a platinum microdisk (radius = 0.25 mm). The platinum electrode was polished prior to use in a rotatory plate. Then it was rinsed with acetone and dried with a tissue. For the electropolymerization, the working electrode was an ITO coated glass (active surface = 1 cm^2^). The ITO/glass electrode was sonicated in acetonitrile, then rinsed with ethanol, and allowed to dry at room temperature in a dust-free atmosphere before using it. The monomer solution concentration used during electrochemical studies was typically 5 × 10^−3^ M. The electrolyte solution consisted of 0.10 M Bu_4_NPF_6_ (electrochemical grade from Fluka) in anhydrous CH_2_Cl_2_ (Aldrich 99.8%, <0.001% H_2_O). CH_2_Cl_2_ (HPLC grade) was used as received and transferred via a syringe under nitrogen. All the solutions were degassed with high purity argon, which was pre-saturated with the solvent before entering the cell. The useful potential range for the electrolytic medium was from 2 V to −2.5 V. Ferrocene (Fc) was employed as an internal reference to ensure the accuracy of the calomel reference electrode. The Fc+/Fc couple in CH_2_Cl_2_ was regularly checked, and a constant value of 0.45 V/SCE was measured. All potentials were referenced relative to the saturated calomel electrode.

### 3.4. Synthesis of the Monomers

#### General Synthesis

The appropriate thiophene alcohol (0.82 mmol), the selected carboxylic acid (1.23 mmol), DCC (2.46 mmol) and DMAP (2.05 mmol) were dissolved in CH_2_Cl_2_ (15 mL) at 0 °C for 30 min. The resulting mixture was stirred at room temperature for 12 h under inert atmosphere. The suspension was filtered in order to remove DCU formed during the reaction, and the filtrates were concentrated under reduced pressure at 45 °C. The crude product was purified by column chromatography in silica gel, using *n*-hexane/CH_2_Cl_2_ (2:5) and then pure CH_2_Cl_2_ as eluent to give the desired product **TPM1** (Figure 1). All monomers were characterized by ^1^H- and ^13^C-NMR spectroscopy and the spectra are included in the Supplementary Materials.

*2-(Thiophen-3-yl)ethylpyrene-1-carboxylate* (**TPM1**): 3-Thiopheneethanol (0.105 g, 0.82 mmol), 1-pyrenecarboxylic acid (0.302 g, 1.23 mmol), DCC (0.507 g, 2.46 mmol) and DMAP (0.250 mg, 2.05 mmol). Light yellow powder. Yield: 87%. MS-CI: *m*/*z* = 356.0. ^1^H-NMR (CDCl_3_, ppm) (Scheme S1) δ: 7.85–9.2 (m, 9H, Py), 7.37 (dd, 1H, H^5^, *J* = 4.9, 3.0 Hz), 7.21 (d, 1H, H^2^, *J* = 4.7 Hz), 7.15 (dd, 1H, H^4^, *J* = 4.9, 1.3 Hz), 4.76 (t, 2H, *J* = 6.8 Hz), 3.26 (t, 2H, *J* = 6.8 Hz). ^13^C-NMR (CDCl_3_, ppm) (Scheme S1-1): 168.09 (C=O), 155.31 (C^2^, Thioph), 138.38 (C^3^, Thioph), 117.89 (C^4^, Thioph), 96.24 (C^1^, Thioph), 134.41, 131.19, 131.09, 130.46, 129.72, 129.51, 128.47, 127.25, 126.40, 126.38, 126.27, 125.91, 124.98, 124.24, 123.71, 121.94 (C_Py_), 65.23 (OCH_2_), 29.94 (CH_2_).

*2-(Thiophen-3-yl)ethyl 4-(pyren-1-yl)butanoate* (**TPM2**): 3-Thiopheneethanol (0.105 g, 0.82 mmol), 1-pyrenebutyric acid (0.355 g, 1.23 mmol), DCC (0.507 g, 2.46 mmol) and DMAP (0.250 mg, 2.05 mmol). White powder. Yield: 82%. MS-CI: *m*/*z* = 398.1. ^1^H-NMR (CDCl_3_, 300 MHz, ppm) (Scheme S2) δ (ppm): 7.82–8.32 (m, 9H, Py), 7.23 (dd, 1H, H^5^, *J* = 4.7, 3.1 Hz), 7.01 (d, 1H, H^2^, *J* = 4.6 Hz), 6.95 (dd, 1H, H^4^, *J* = 4.9, 1.1 Hz), 4.31 (t, 2H, H^β^, *J* = 7.0 Hz), 3.37 (t, 2H, H^α^, *J* = 6.6 Hz), 2.96 (t, 2H, H^6^, *J* = 6.9 Hz), 2.49 (t, 2H, H^8^, *J* = 7.2 Hz), 2.09–2.30 (m, 2H, H^7^). ^13^C-NMR (CDCl_3_, 75 MHz, ppm) (Scheme S2-1): 174.17 (C=O, C_a_), 155.30 (C^2^, Thioph), 138.28 (C^3^, Thioph), 124.57 (C^4^, Thioph), 116.61 (C^1^, Thioph), 137.38, 136.38, 130.04, 126.81, 126.47, 126.16, 125.81, 125.57, 125.54, 124.98, 124.57, 124.24, 122.71, 121.74 (C_Py_), 63.02 (OCH_2_, C_b_), 36.17, 34.38, 30.45, 23.47 (CH_2_, C_c_), 33.36 (Py-CH_2_).

*4-(Pyren-1-yl)butyl 2-(thiophen-3-yl)acetate* (**TPM3**): 3-Thiopheneacetic acid (0.110 g, 0.78 mmol), 1-pyrenebutanol (0.224 g, 0.82 mmol), DCC (0.507 g, 2.46 mmol) and DMAP (0.250 mg, 2.05 mmol). Light yellow powder. Yield: 76%, MS-CI: *m*/*z* = 398.1. ^1^H-NMR (CDCl_3_, 300 MHz, ppm) (Scheme S3) δ (ppm): 7.85–8.3 (m, 9H, Py), 7.24 (dd, 1H, H^5^, *J* = 4.9, 3.1 Hz), 7.12 (d, 1H, H^2^, *J* = 4.7 Hz), 7.00 (d, 1H, H^4^, *J* = 4.9, 1.3 Hz), 4.15 (dt, 2H, *J* = 14.3, 6.8 Hz), 3.64 (s, 2H, H^α^), 3.36 (t, 2H, H^6^, *J* = 7.5 Hz), 1.87 (m, 2H, H^8^), 1.27 (t, 2H, H^9^, *J* = 7.1 Hz).^13^C-NMR (CDCl_3_, 75 MHz, ppm) (Scheme S3-1): 171.28 (C=O, C_a_), 156.21 (C^2^, Thioph), 136.38 (C^3^, Thioph), 133.77 (C^4^, Thioph), 115.52 (C^1^, Thioph), 131.55, 131.00, 129.99, 128.71, 128.57, 127.61, 127.41, 127.33, 126.76, 125.95, 125.79, 125.14, 125.02, 124.91, 124.84, 123.37 (C_Py_), 64.85 (OCH_2_, C_b_), 36.04, 33.09, 28.69, 28.15 (CH_2_).

*Pyren-1-ylmethyl 2-(thiophen-3-yl)acetate* (**TPM4**): 3-Thiopheneacetic acid (0.110 g, 0.78 mmol), 1-pyrenemethanol (0.190 g, 0.82 mmol), DCC (0.507 g, 2.46 mmol) and DMAP (0.250 mg, 2.05 mmol). Beige powder. Yield: 65%, MS-CI: *m*/*z* = 356. ^1^H-NMR (CDCl_3_, 300 MHz, ppm) (Scheme S4): δ (ppm): 8.02–8.24 (m, 9H, Py), 7.23 (dd, 1H, H^5^, *J* = 4.6, 3.1 Hz), 7.15 (d, 1H, H^2^, *J* = 3.2 Hz), 7.04 (dd, 1H, H^4^, *J* = 4.9, 1.3 Hz), 5.85 (s, 2H, H^6^), 3.73 (s, 2H, H^α^). ^13^C-NMR (CDCl_3_, 75 MHz, ppm) (Scheme S4-1): 168.51 (C=O, C_a_), 151.41 (C^2^, Thioph), 138.44 (C^3^, Thioph), 136.26 (C^4^, Thioph), 120.74 (C^1^, Thioph), 132.94, 132.26, 131.60, 130.04, 129.59, 129.17, 127.72, 126.90, 126.81, 126.10, 124.90, 123.81, 122.52, 12.41, 121.75 (C_Py_), 64.70 (OCH_2_, C_b_), 36.17 (CH_2_, C_c_), 32.00 (Py-CH_2_, C_d_).

*4-(Pyren-1-yl)butyl thiophene-3-carboxylate* (**TPM5**): 1-Pyrenecarboxylic acid (0.157 g, 1.22 mmol), 1-pyrenebuthanol (0.224 g, 0.82 mmol), DCC (0.507 g, 2.46 mmol) and DMAP (0.250 mg, 2.05 mmol). White powder. Yield: 79%, MS-CI: *m*/*z* = 384.1. ^1^H-NMR (CDCl_3_, 300 MHz, ppm) (Scheme S5) δ (ppm): 7.80–8.23 (m, 9H, Py), 7.51 (dd, 1H, H^5^, *J* = 5.1, 1.2 Hz), 7.28 (d, 1H, H^2^, *J* = 3.1 Hz), 7.02 (d, 1H, H^4^, *J* = 4.9 Hz), 4.36 (t, 2H, H^6^, *J* = 6.4 Hz), 3.41 (t, 2H, H^9^ *J* = 7.1 Hz), 1.75–2.17 (m, 4H, H^7^, H^8^). ^13^C-NMR (CDCl_3_, 75 MHz, ppm) (Scheme S5-1): 162.94 (C=O, C_a_), 151.51 (C^2^, Thioph), 136.40 (C^3^, Thioph), 133.94 (C^4^, Thioph), 100.01 (C^1^, Thioph), 131.55, 131.00, 129.99, 128.71, 128.00, 127.61, 127.59, 127.40, 127.35, 125.93, 125.22, 125.01, 124.91, 124.83, 123.83 (C_Py_), 64.57 (OCH_2_, C_b_), 33.14, 28.79, 28.21 (CH_2_).

### 3.5. Synthesis of the Oligomers and the Polymers

For the obtainment of the pyrene containing polythiophenes, chemical and electrochemical polymerization methods were employed. When a chemical polymerization was carried out only oligomers were obtained; this was confirmed by MALDI-TOF spectrometry (Schemes S11–S15).

#### 3.5.1. Chemical Polymerization

All the oligomers (Figure 2) were obtained according to the procedure previously reported in the literature [18]. The appropriate TPM monomer (0.216 mmol) was dissolved in dry CHCl_3_ (20 mL) and the solution was stirred for 10 min under inert atmosphere. Afterwards, a solution of FeCl_3_ (0.864 mmol) in CHCl_3_ (10 mL) was prepared and added dropwise to the monomer solution; then it was stirred for 12 h. After this time, cold methanol (20 mL) was added to the reaction mixture, which was poured into ice-water (60 mL) in order to precipitate the polymer. The crude product was separated by filtration, washed with cold methanol and treated with NH_4_OH 20% in order to reduce the crude product, because the polymer was obtained in the oxidized form. Finally, the polymer was purified by Soxhlet extraction, using a mixture of methanol:water 8:2. After 12 h, the mixture of solvents was changed by pure acetone, and the compound was extracted for 12 more hours. The final wash was carried out with cold pure methanol (0–5 °C) and the compound was filtered in a Buchner under vacuum to give the desired TPO oligomer (Figure 2).

#### 3.5.2 Synthesis of **TPO1**–**5**

**TPM1** (0.077 g, 0.216 mmol) FeCl_3_ (0.14 g, 0.864 mmol). Oligomer **TPO1** (Figure 2): orange powder. Yield: 71%. MALDI-TOF^+^; homopolymers, *m*/*z* = 1064.5 (*n* = 3), 1418.2 (*n* = 4), 1772.1 (*n* = 5), 2126.4 (*n* = 6) (Scheme S11).

**TPM2** (0.216 mmol) FeCl_3_ (0.14 g, 0.864 mmol). **TPO2** (Figure 2): light orange powder. Yield: 68%. MALDI-TOF^+^; homopolymers: *m*/*z* = 1190.4 (*n* = 3), 1586.6 (*n* = 4), 1982.9 (*n* = 5), 2379.1 (*n* = 6), 2774.3 (*n* = 7), 3171.4 (*n* = 8) (Scheme S12).

**TPM3** (0.086 g, 0.216 mmol), FeCl_3_ (0.14 g, 0.864 mmol). **TPO3** (Figure 2): brown powder. Yield: 74%. MALDI-TOF^+^; homopolymers, *m*/*z* = 1190.5 (*n* = 3), 1586.7 (*n* = 4), 1983.0 (*n* = 5), 2379.3 (*n* = 6) (Scheme S13).

**TPM4** (0.077 g, 0.216 mmol), FeCl_3_ (0.14 g, 0.864 mmol). **TPO4** (Figure 2): brown solid. Yield: 46%. MALDI-TOF^+^; homopolymers, *m*/*z* = 1185.7 (*n* = 3), 1418.7 (*n* = 4), 1772.6 (*n* = 5) (Scheme S14).

**TPM5** (0.083 g, 0.216 mmol), FeCl_3_ (0.14 g, 0.864 mmol). **TPO5** (Figure 2): dark brown powder. Yield: 69%. MALDI-TOF^+^; homopolymers, *m*/*z* = 766.3 (*n* = 2), 1148.4 (*n* = 3), 1530.5 (*n* = 4), 1912.5 (*n* = 5), 2294.2 (*n* = 6), 2676.4 (*n* = 7), 3058.3 (*n* = 8) (Scheme S15).

### 3.6. Thermal Analysis

The thermogravimetric analysis (TGA) was determinate and conducted with samples of 5 mg on a with a heating rate of 10 °C/min. The melting point and the glass transition temperature (T_g_) were measured with samples of 5–10 mg, from −50 to 250 °C, with a heating rate of 5 °C/min, under N_2_ flux of 50 cc/min, with a period of 40 s and an amplitude of 0.5 °C. (Schemes S6–S10).

## 4. Conclusions

A new series of monomers, oligo and polythiophenes bearing pyrene units and flexible alkyl spacers were synthesized and characterized. These novel compounds exhibited a good thermal stability with T_10_ values between 256 and 299 °C and Tg values varying from 36 to 39 °C. Except for monomer **TPM1**, which exhibited an absorption band at 353 nm, the other monomers and polymers exhibited absorption bands at λ = 345 nm due to S_0_ → S_2_ transition of the pyrene groups. The fluorescence spectra of the obtained polymers showed two emission bands, a “monomer” emission at λ = 390–410 nm, followed by an intense excimer emission at λ = 539–545 nm, which reveals the presence of intramolecular pyrene-pyrene interactions. We found that all the monomer may be electropolymerized to generate electroactive thin films deposited onto ITO. These films were constituted of polythiophene chains with pending pyrene units, which did not participate in the electropolymerization process. These materials can be held in their oxidized or neutral state and the polymerization occurs exclusively in the thiophene rings. Moreover, the pyrene units can act as a fluorescent probe or anchor to other conjugated materials such as graphene by π–π interactions, which make these materials very promising for a wide variety of applications in the field of organic electronics.

## Supplementary Materials

Supplementary materials can be accessed at: http://www.mdpi.com/1420-3049/21/2/172/s1.
