# Supplementary material for: Synthesis and Characterization of Novel Polythiophenes Containing Pyrene Chromophores: Thermal, Optical and Electrochemical Properties
Source: Molecules. 2016 Jan 30;21(2):172. doi: 10.3390/molecules21020172 (PMC6273483; doi:10.3390/molecules21020172)
Supplement: Supplementary file 1 [file molecules-21-00172-s001.pdf]

Molecules **2016**, *21*, 172; doi:10.3390/molecules21020172

Supplementary Materials: Synthesis and Characterization of Novel Polythiophenes Containing Pyrene Chromophores: Thermal, Optical and Electrochemical Properties

Bianca X. Valderrama-García, Efraín Rodríguez-Alba, Eric G. Morales-Espinoza, Kathleen Moineau Chane-Ching and Ernesto Rivera

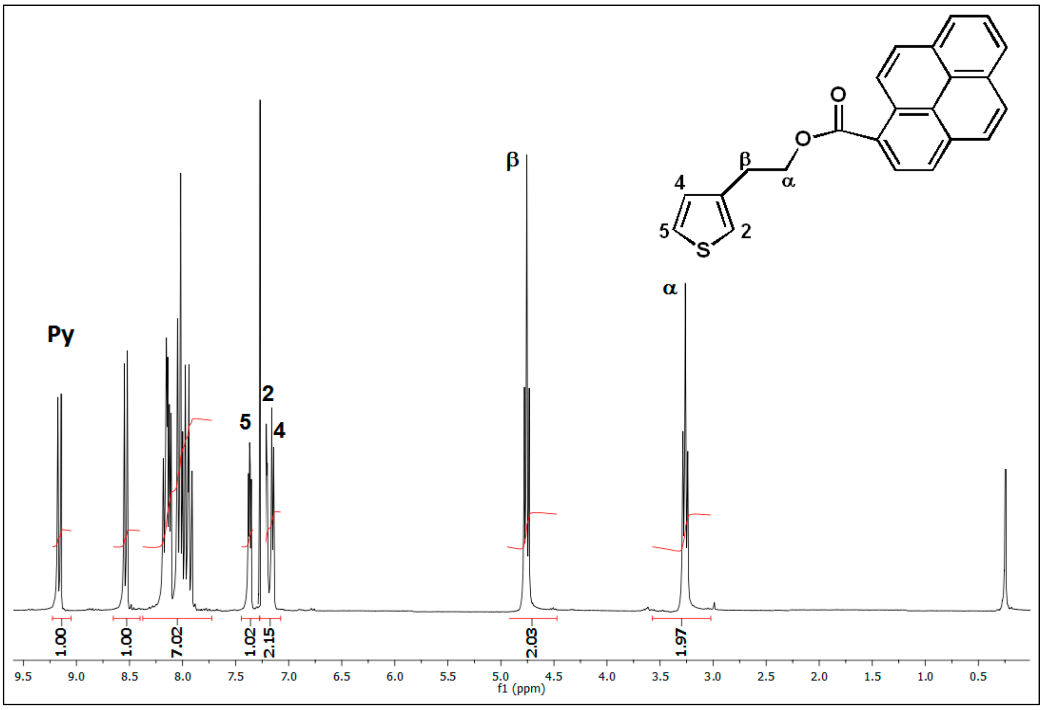

Scheme S1. ^{1}H-NMR spectrum of **TPM1** in $CDCl_3$ solution.

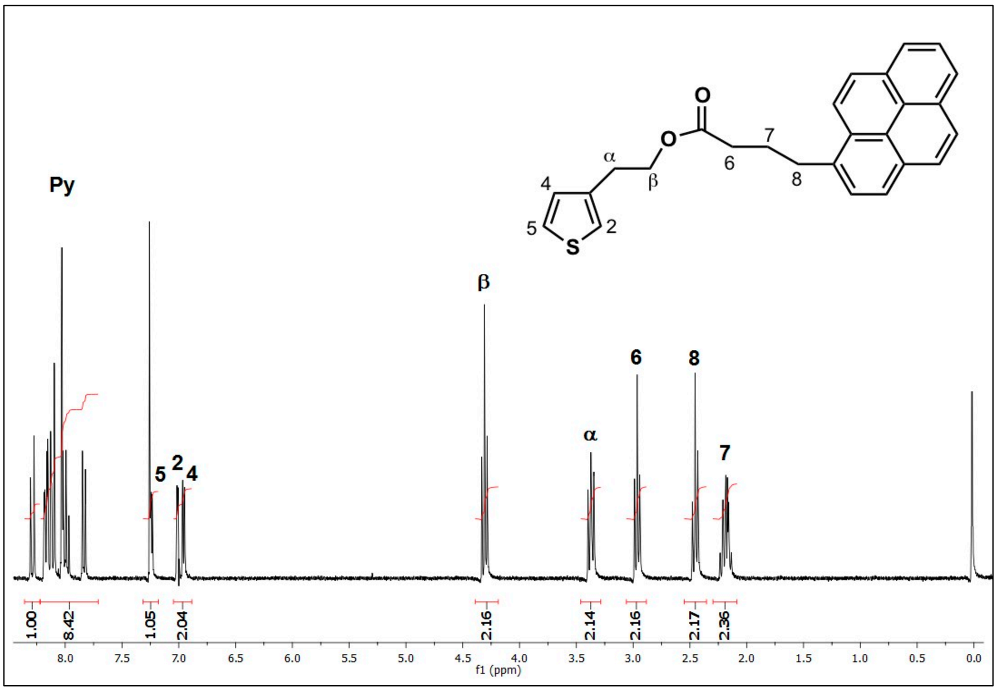

Scheme S2. ^{1}H-NMR spectrum of **TPM2** in $CDCl_3$ solution.

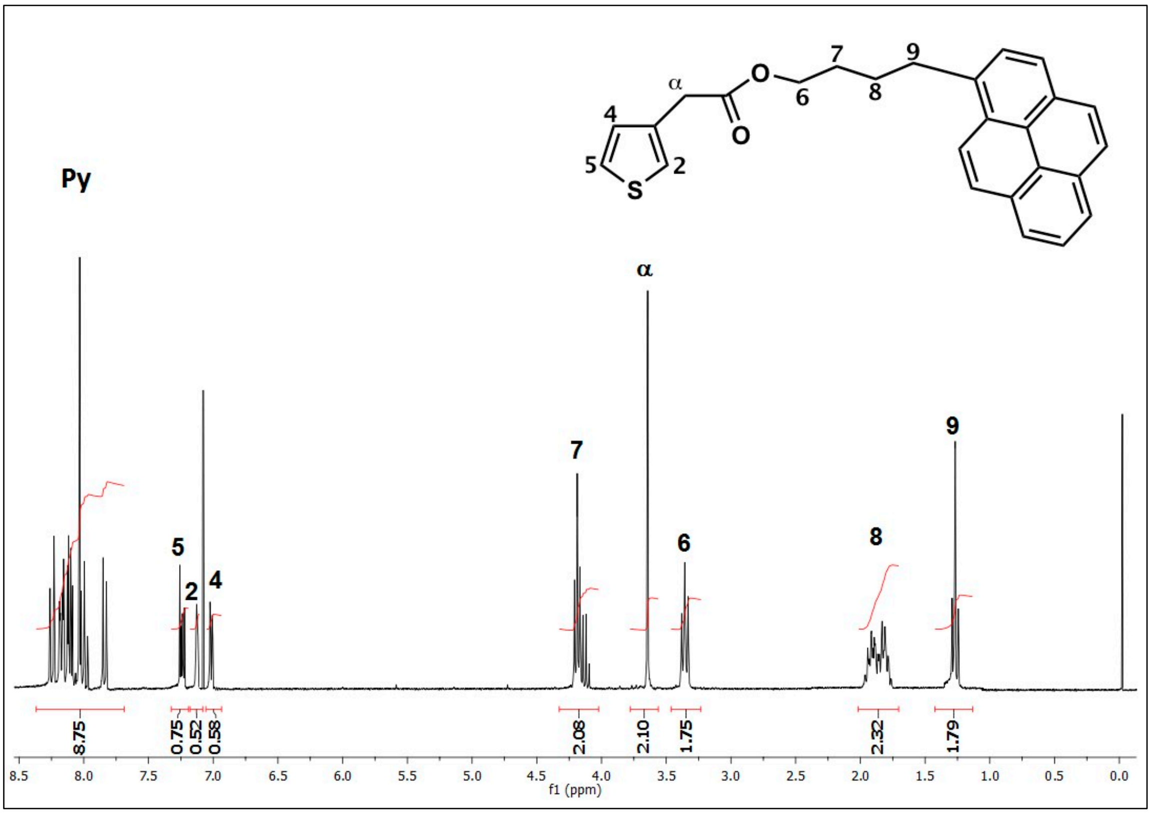

Scheme S3. ^{1}H-NMR spectrum of **TPM3** in $CDCl_3$ solution.

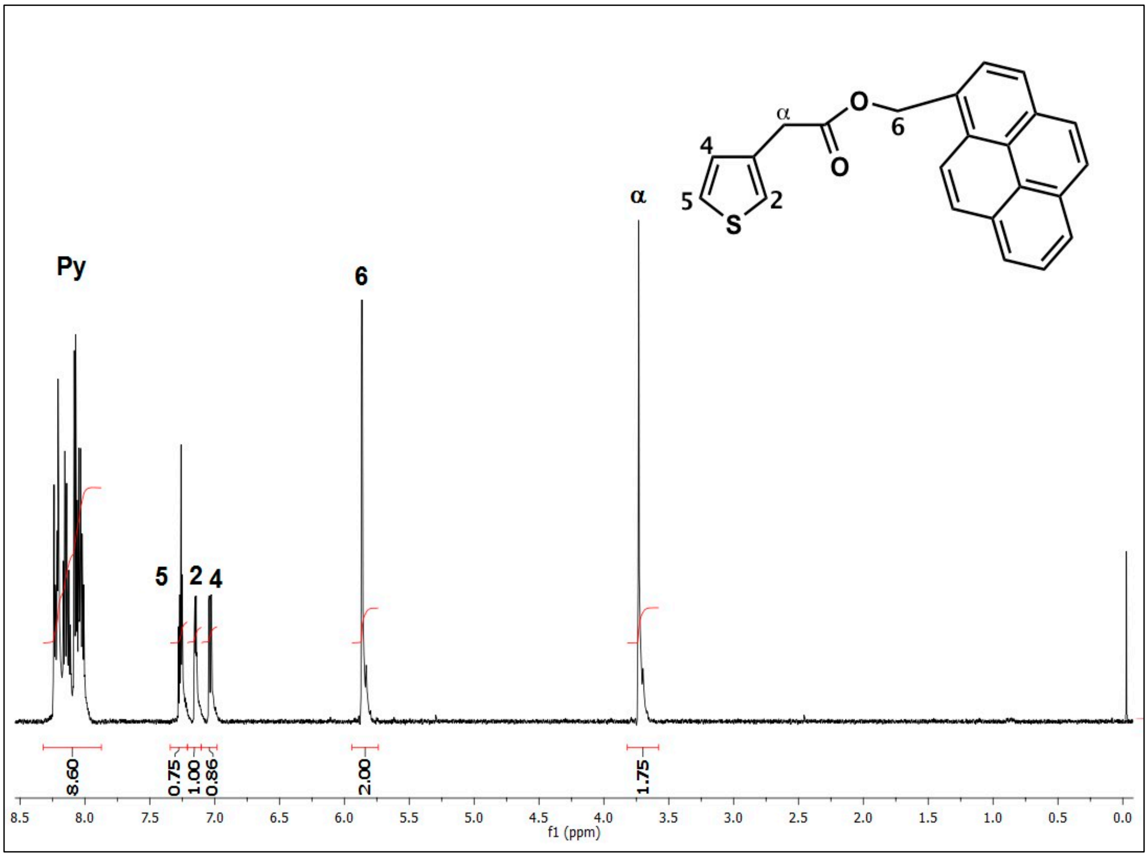

Scheme S4. ^{1}H-NMR spectrum of **TPM4** in $CDCl_3$ solution.

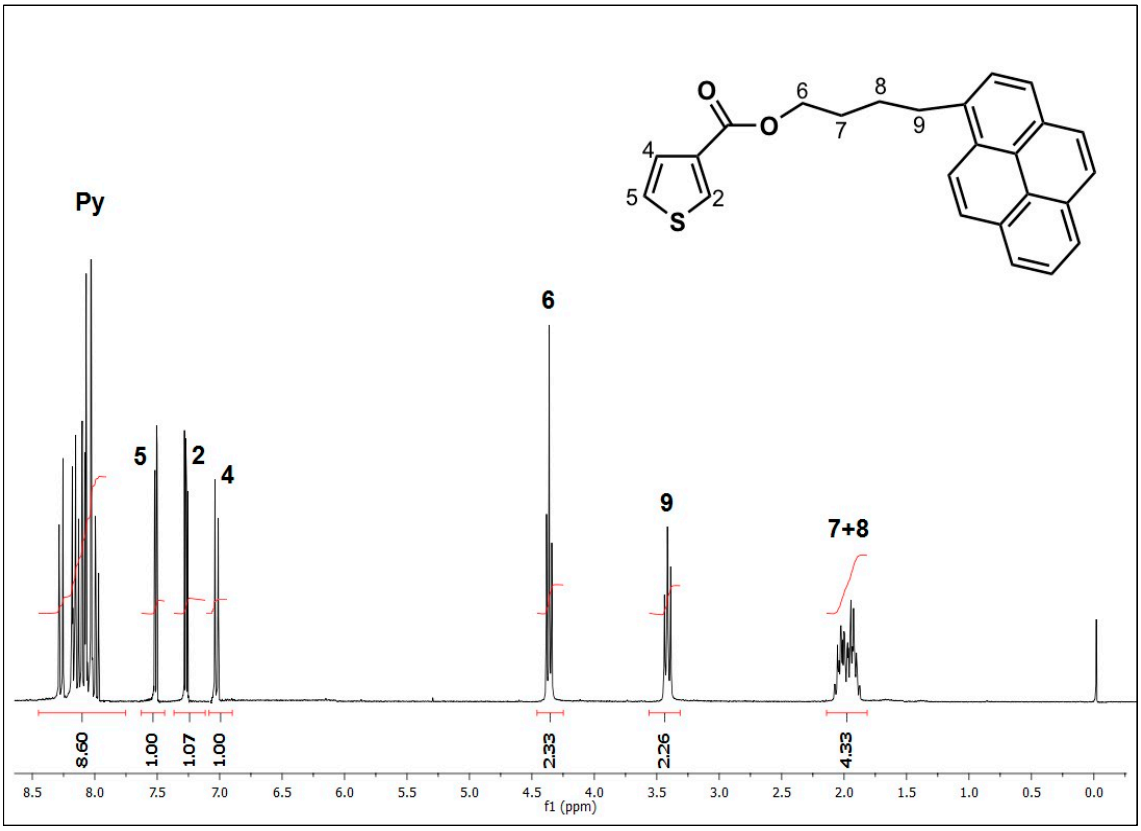

Scheme S5. ^{1}H-NMR spectrum of **TPM5** in $CDCl_3$ solution.

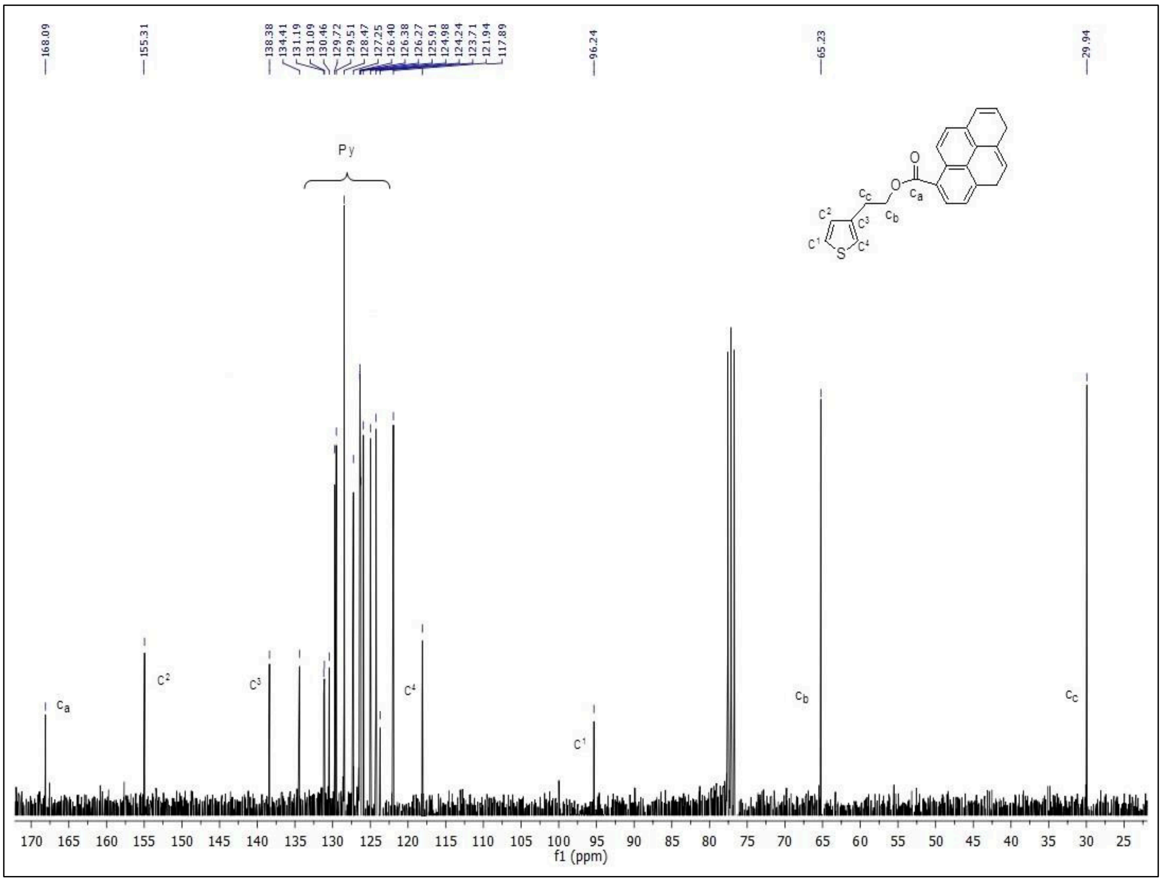

Scheme S1-1. ^{13}C-NMR spectrum of **TPM1** in $CDCl_3$ solution.

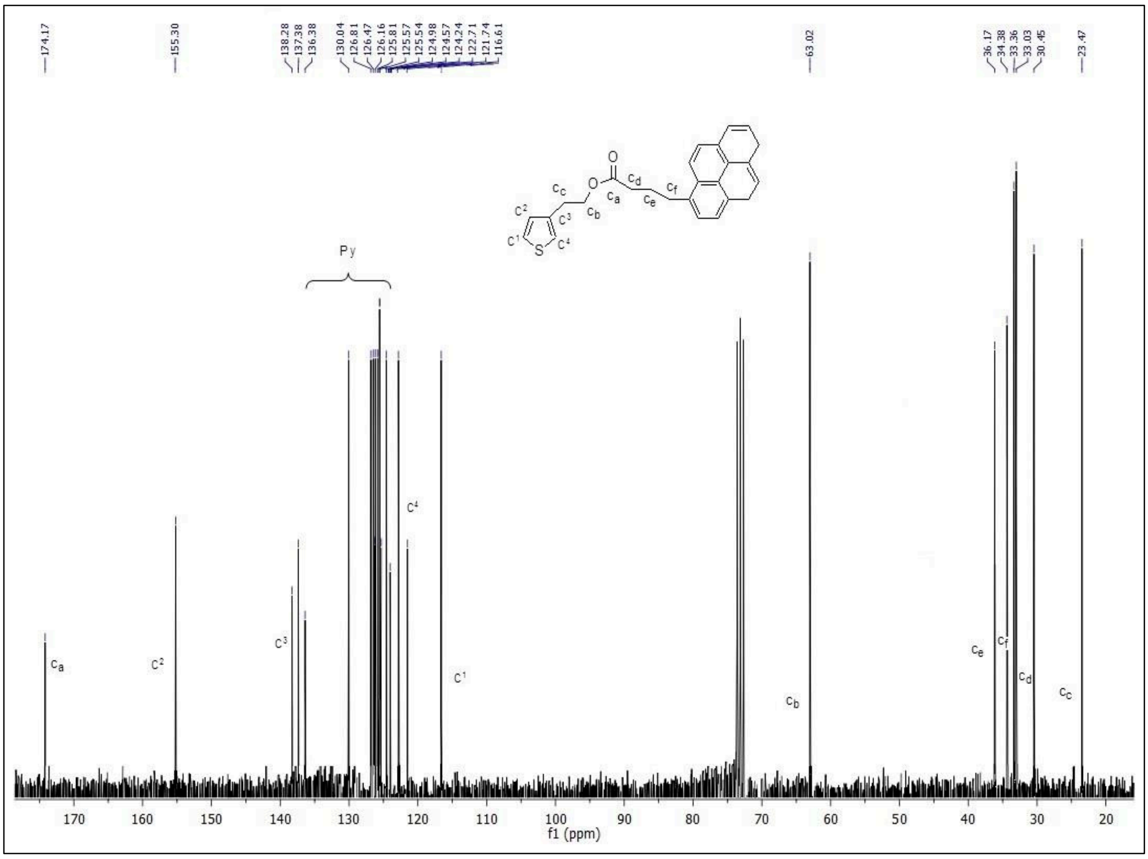

Scheme S2-1. ^{13}C-NMR spectrum of **TPM2** in $CDCl_3$ solution.

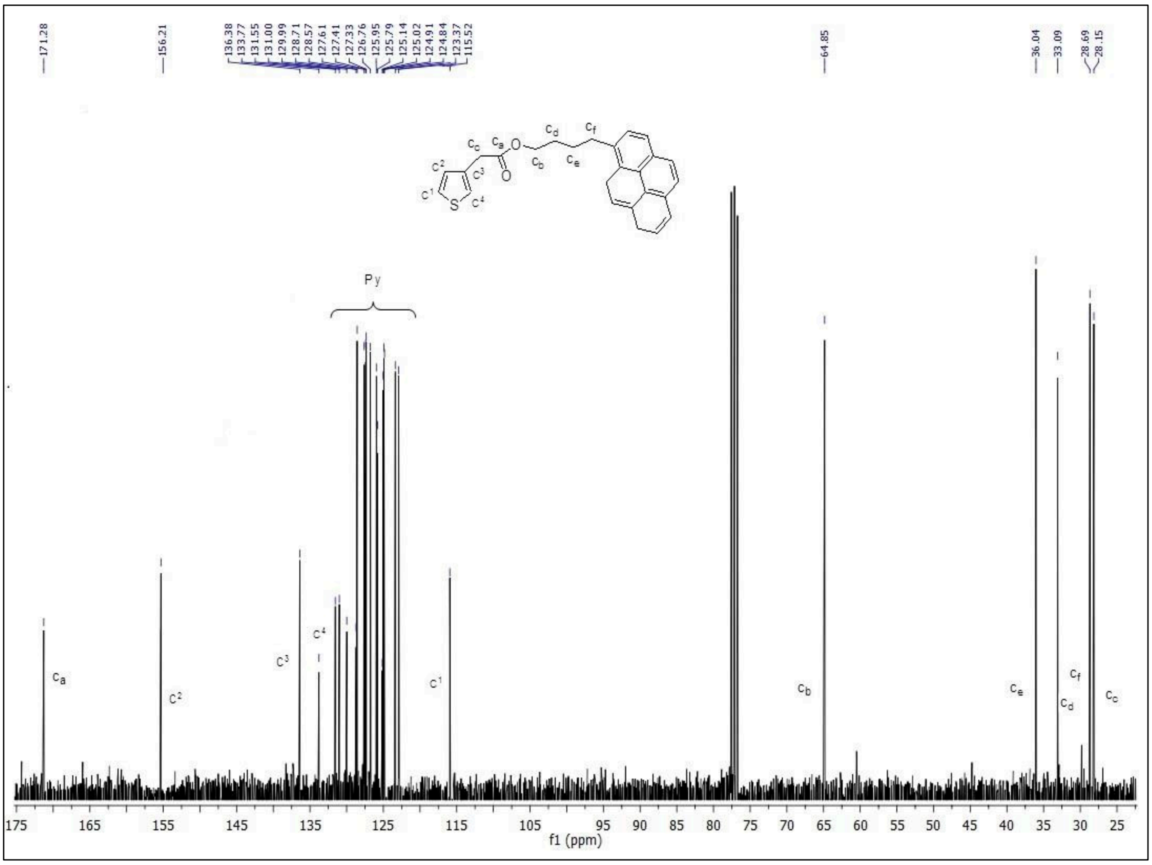

Scheme S3-1. ^{13}C-NMR spectrum of **TPM3** in $CDCl_3$ solution.

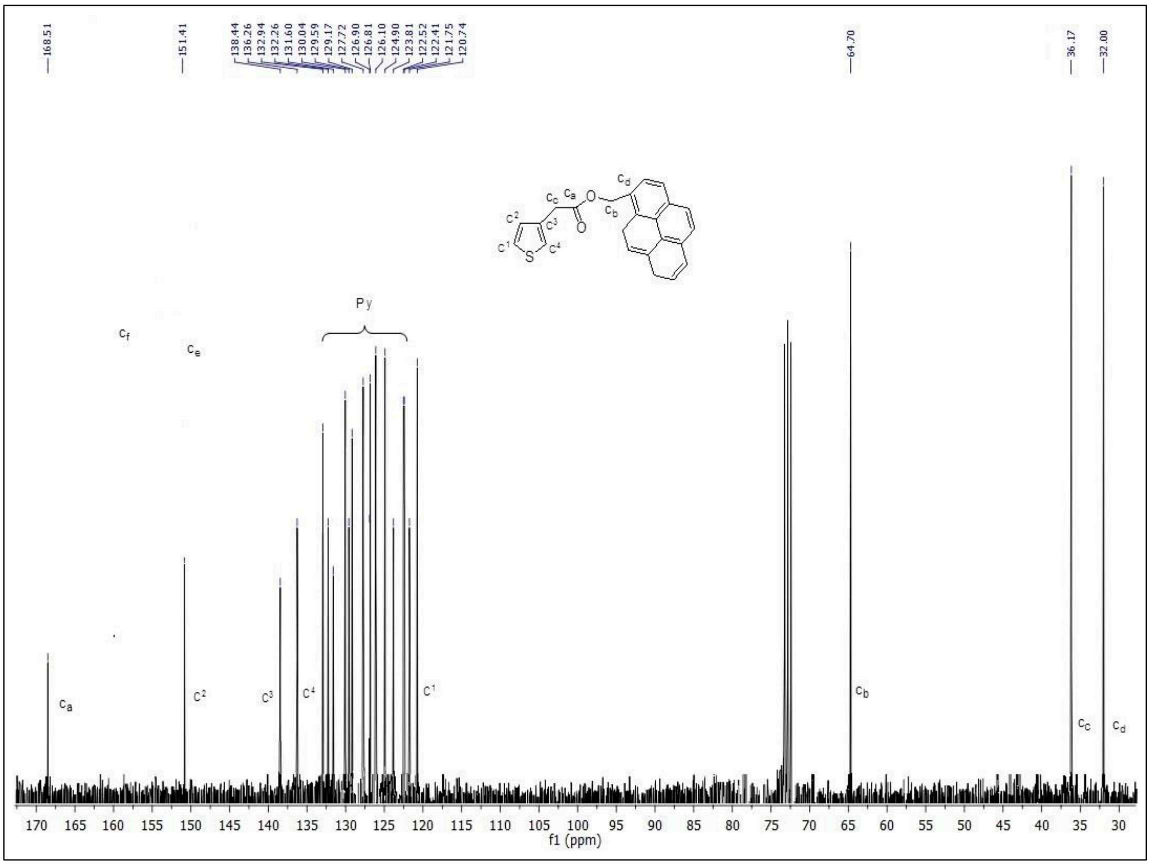

Scheme S4-1. ^{13}C-NMR spectrum of **TPM4** in $CDCl_3$ solution.

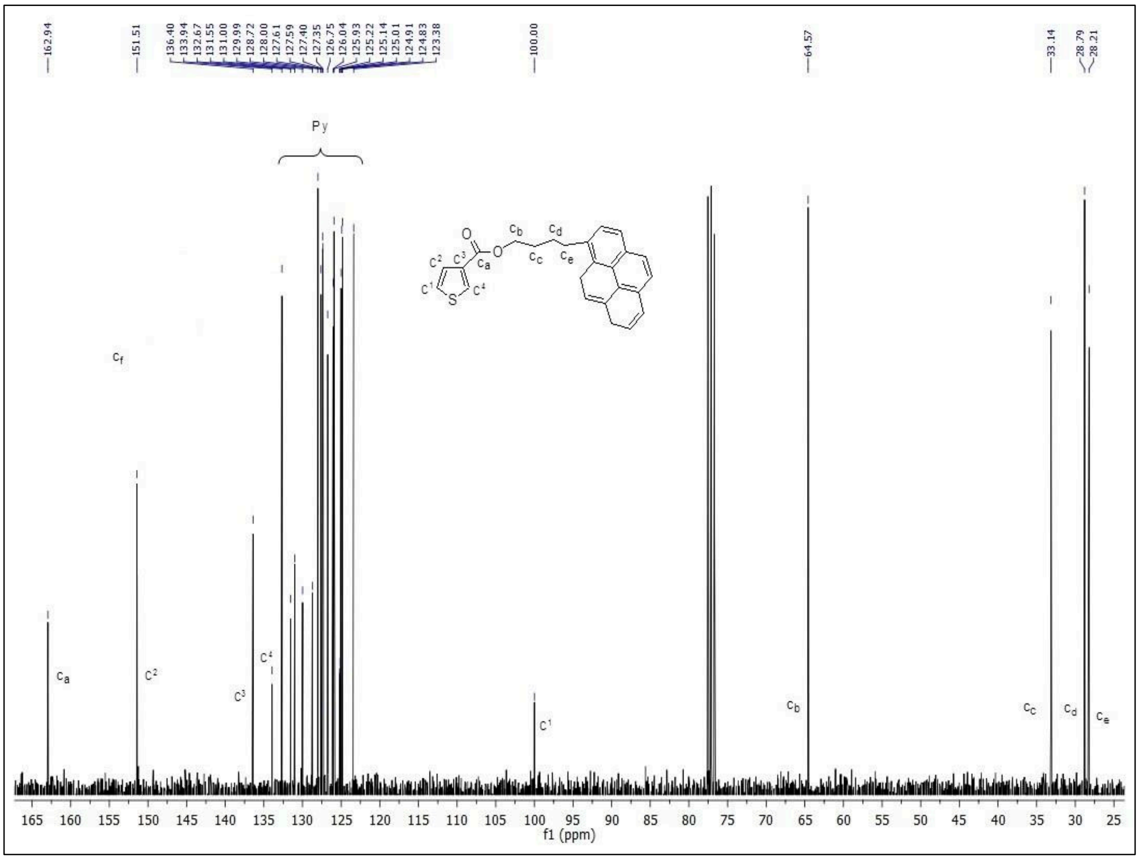

Scheme S5-1. ^{13}C-NMR spectrum of **TPM5** in $CDCl_3$ solution.

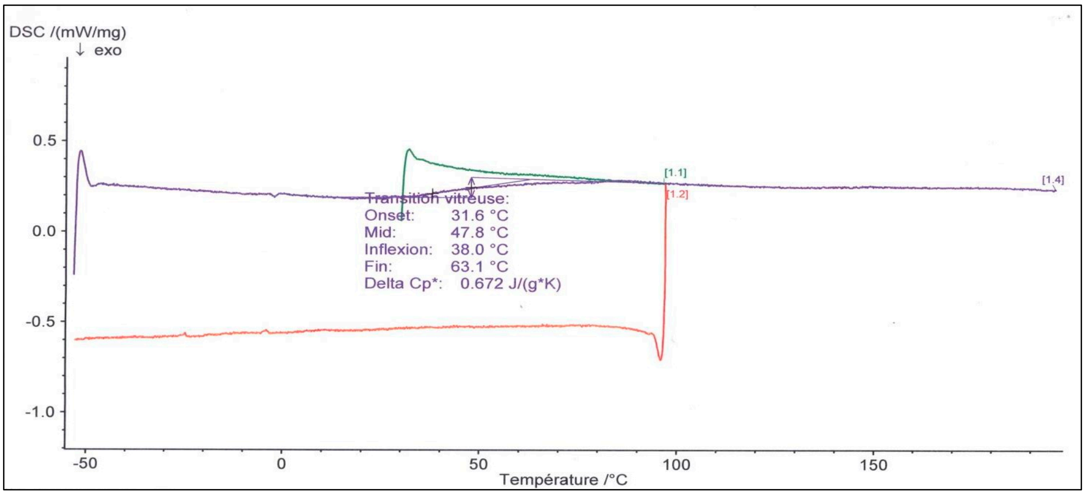

Scheme S6. DSC of TPO1.

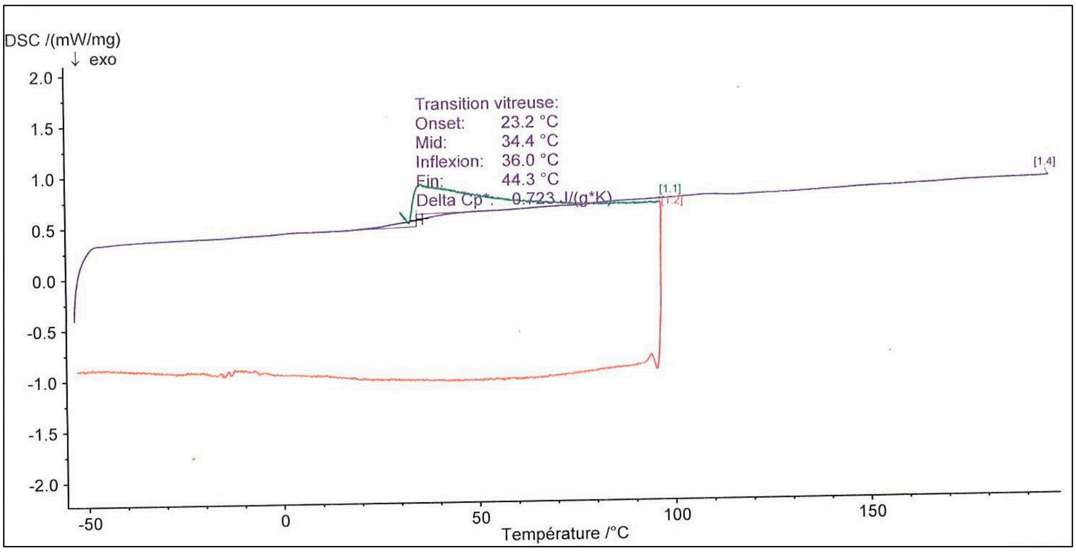

Scheme S7. DSC of TPO2.

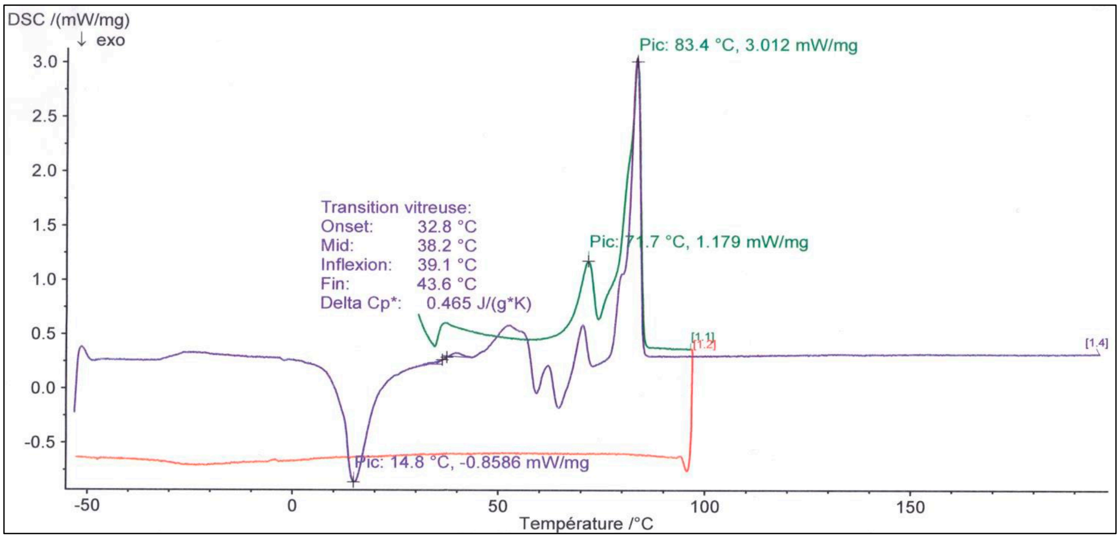

Scheme S8. DSC of TPO3.

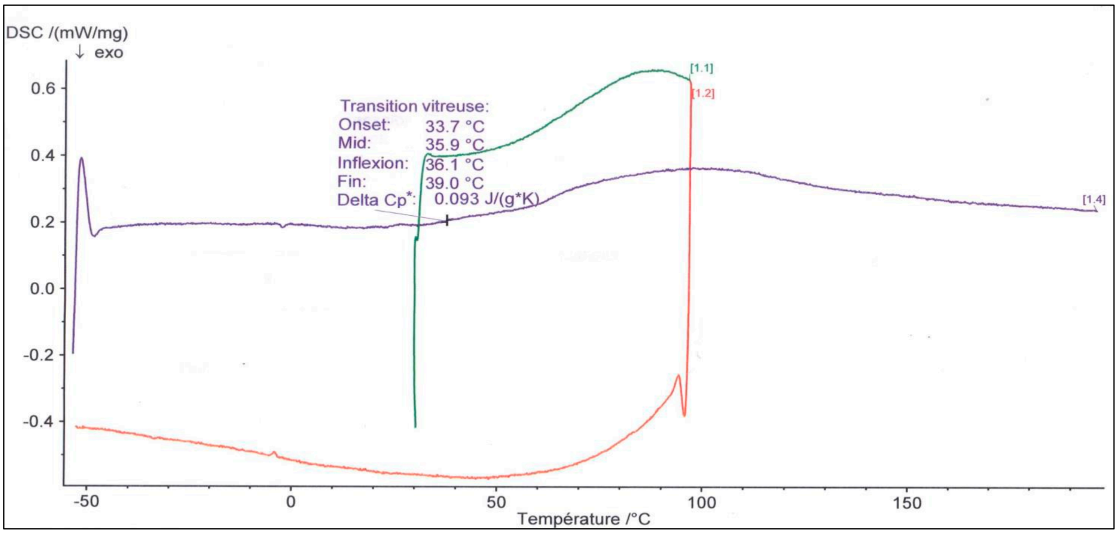

Scheme S9. DSC of TPO4.

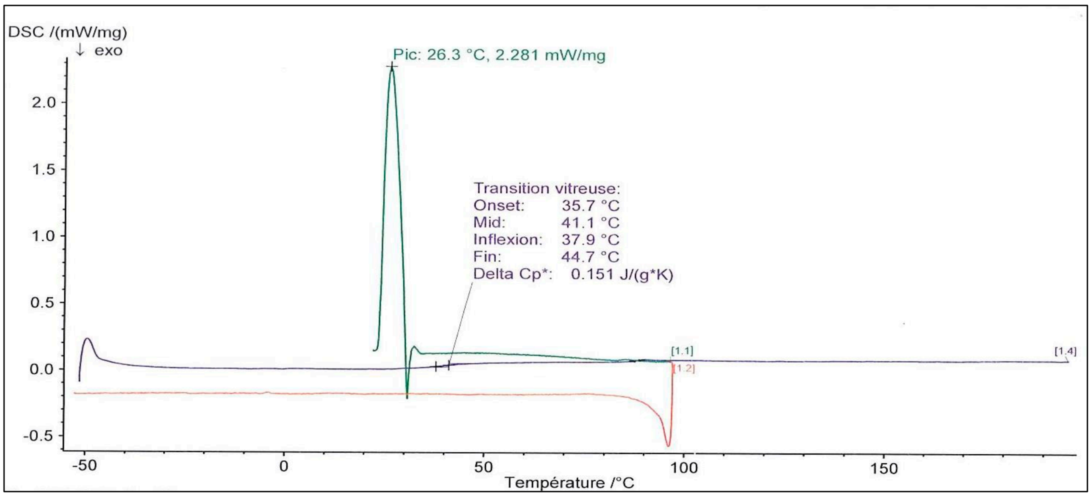

Scheme S10. DSC of TPO5.

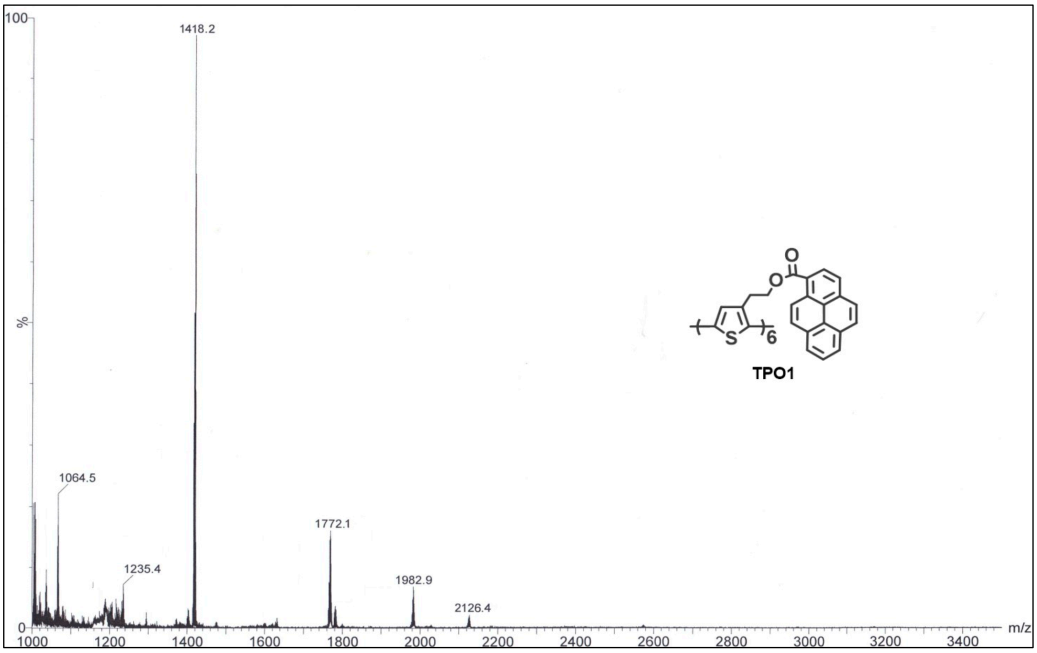

Scheme S11. MALDI-TOF of TPO1.

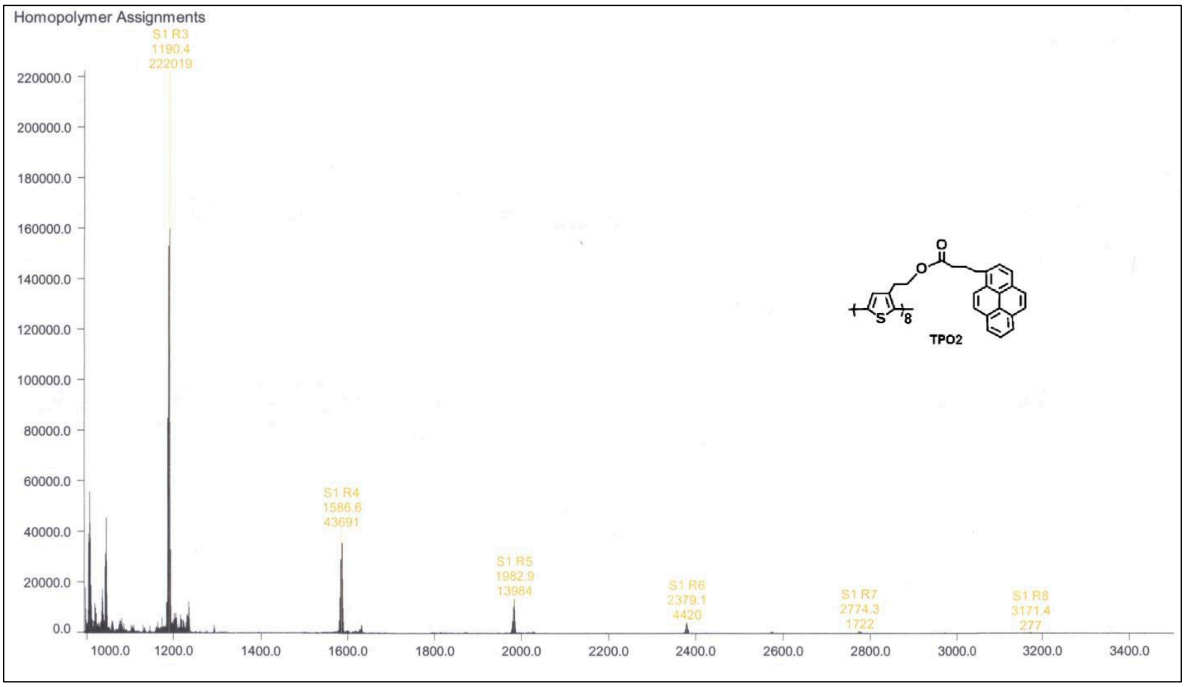

Scheme S12. MALDI-TOF of TPO2.

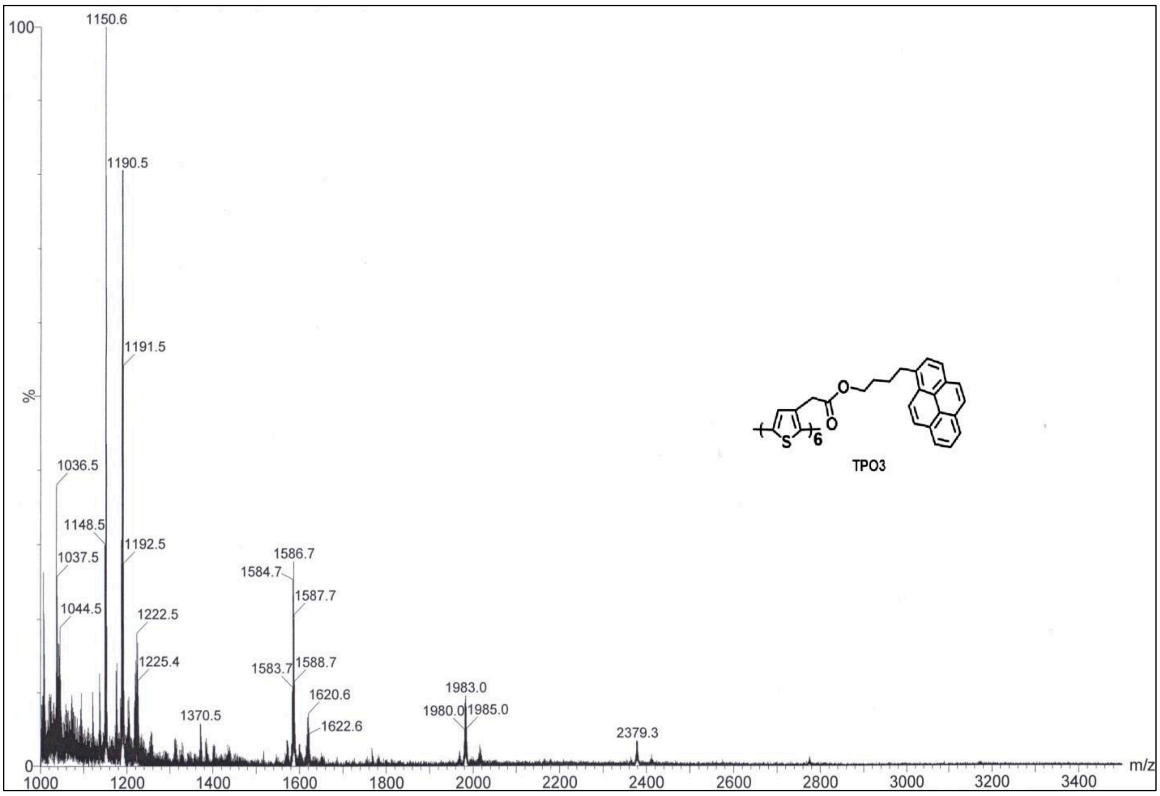

Scheme S13. MALDI-TOF of TPO3.

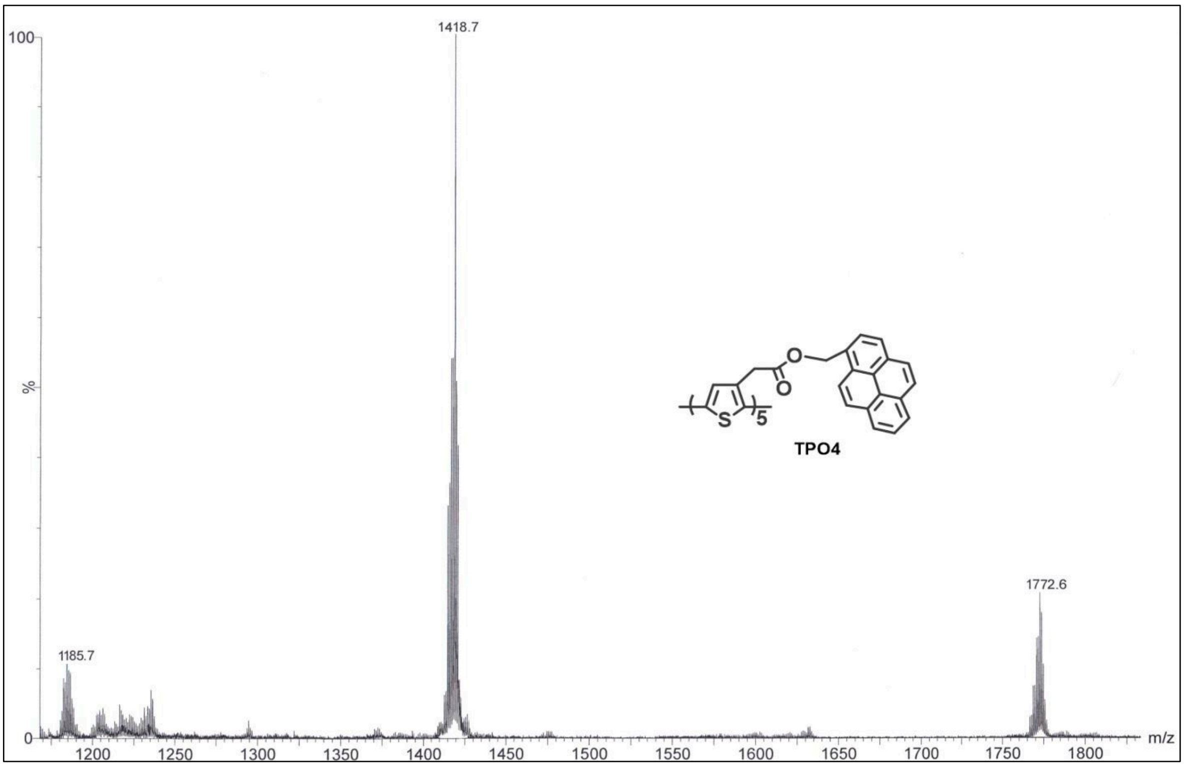

Scheme S14. MALDI-TOF of TPO4.

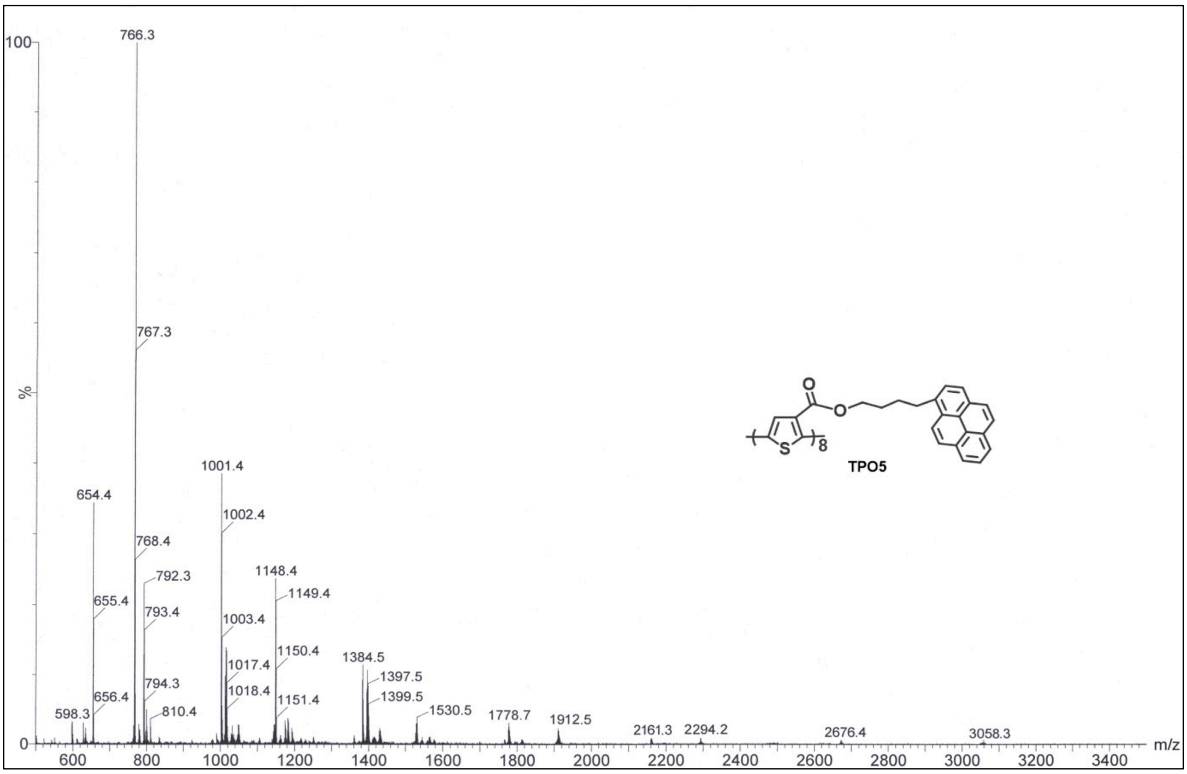

Scheme S15. MALDI-TOF of TPO5.
